# TGF-β1-triggered maladaptive bone marrow endothelium impedes hematopoietic recovery

**DOI:** 10.1038/s41392-025-02429-y

**Published:** 2025-10-07

**Authors:** Zhong-Shi Lyu, Meng-Zhu Shen, Yuan-Ya Zhang, Hui Gao, Mi Liang, Yu-Hong Chen, Zhen-Kun Wang, Xin-Yan Zhang, Dan-Dan Chen, Yuan-Yuan Zhang, Meng Lv, Xiao-Do Mo, Lan-Ping Xu, Xiao-Hui Zhang, Yu Wang, Ying-Chun Wang, Yuan Kong, Xiao-Jun Huang

**Affiliations:** 1 Peking University People’s Hospital, Peking University Institute of Hematology, National Clinical Research Center for Hematologic Disease, Beijing Key Laboratory of Cell and Gene Therapy for Hematologic Malignancies, Peking University, Beijing, China; 2 Peking-Tsinghua Center for Life Sciences, Academy for Advanced Interdisciplinary Studies, Peking University, Beijing, China; 3 Institute of Genetics and Developmental Biology, Chinese Academy of Sciences, Beijing, China; 4 University of Chinese Academy of Sciences, Beijing, China

**Keywords:** Translational research, Haematological diseases

## Abstract

Endothelial cells (ECs) form a critical bone marrow (BM) niche for hematopoietic stem cell regulation in homeostasis and stress states. However, BM ECs are frequently disrupted in hematologic diseases and their clinical interventions. Although transient EC injury is repairable, chronic activation of stress signals often induces maladaptive EC repair, which is a state of fibrotic reprogramming characterized by the loss of BM EC-specific functions and impaired hematopoietic-supporting ability. Although TGF-β1 (a pleiotropic cytokine) is implicated in angiogenesis and tissue repair, its role in driving BM EC maladaptation remains undefined. Here, in vitro experiments combined with a mouse model with adeno-associated virus-mediated BM EC-specific overexpression of constitutively active TGF-βRI demonstrated that TGF-β1 activation drives maladaptive BM ECs, characterized by defective vascular regeneration and impaired hematopoietic-supporting abilities. Multiomics profiling (transcriptomic and phosphoproteomic analyses) demonstrated a mechanistic link between maladaptive BM EC repair and a TGF-β1-induced secretome shift, characterized by the suppression of hematopoietic-supportive factors and the upregulation of epithelial-mesenchymal transition mediators. This effect may be driven by dysregulated vascular endothelial growth factor receptor/Notch crosstalk and subsequent p38α activation. Clinically, BM ECs from poor graft function (PGF) patients post-transplantation exhibited hyperactivated TGF-β1 signaling. In vitro experiments revealed TGF-β1 inhibition restored the function of maladaptive BM ECs from PGF patients. Subsequently, a prospective single-arm study involving luspatercept (a TGF-β ligand trap) demonstrated the significant promotion of multilineage hematopoiesis recovery in post-transplantation patients (NCT05629260). Therefore, our findings suggest that TGF-β1 may be a critical driver of BM EC maladaptation and highlight therapeutic TGF-β1 pathway inhibition for hematopoietic regeneration via BM EC remodeling.

## Introduction

The bone marrow (BM) vascular niche is fundamental for maintaining lifelong hematopoiesis and sustaining overall blood system homeostasis. Hematopoietic stem cells (HSCs) inhabit specialized niches where they are supported by a variety of stromal components, among which endothelial cells (ECs) are particularly critical. BM ECs function not only as structural scaffolds but also as active regulators that secrete angiocrine signals influencing HSC quiescence, proliferation, and differentiation.^1–13^ Despite their important functions, BM ECs are vulnerable to pathological insults. They are frequently disrupted in hematologic diseases, such as acute myeloid leukemia (AML), aplastic anemia (AA), and myelodysplastic neoplasm (MDS),^4–7,14–19^ and are also severely damaged by clinical interventions, such as chemotherapy and radiation.^15,20^ Although transient EC injury can often be repaired through physiological regeneration, chronic activation of stress signals—arising from persistent inflammation, oxidative stress, or malignant remodeling—tends to induce maladaptive EC repair. This maladaptive state is characterized by fibroblastic features and the loss of tissue-specific functions.^21,22^ As a result, the BM vascular microenvironment loses its capacity to sustain HSC regeneration, leading to hematopoietic failure, and clinical manifestations such as poor graft function (PGF), which is a significant complication post allogeneic hematopoietic stem cell transplantation (allo-HSCT).^4–7,17,23,24^ Although the clinical relevance is well established, the molecular drivers that determine whether BM EC repair is successful or maladaptive remain incompletely understood.

Under physiological conditions, EC regeneration depends heavily on vascular endothelial growth factor (VEGF)—mediated pathways.^12,13,25–28^ In the BM, VEGF-A and VEGF-C coordinate to rebuild sinusoidal networks and facilitate hematopoietic recovery following myelosuppressive injury. These processes are primarily mediated through VEGF receptor 2 (VEGFR2) - dependent survival signals, which not only enhance endothelial viability but also stimulate the release of stem cell—active cytokines that promote hematopoietic reconstitution.^12,29^ Similar mechanisms are observed in other organs, such as the lung, where VEGF-A facilitates alveolar barrier repair and accelerates vascular stabilization after injury.^30,31^ By contrast, under conditions of persistent or severe stress, ECs deviate from this regenerative program and undergo endothelial – to - mesenchymal transition (EndMT). During EndMT, ECs lose their vascular identity and adopt profibrotic and pro—inflammatory features, thereby exacerbating the tissue remodeling and further impairing hematopoietic support.^22^ While several studies have shed light on VEGF and EndMT—related mechanisms, the molecular determinants that govern successful versus maladaptive endothelial repair in the BM remain poorly characterized. Understanding these determinants is essential, as they may uncover therapeutic targets capable of preventing BM EC dysfunction and restoring the hematopoietic—supportive microenvironment.

Transforming growth factor-beta 1 (TGF-β1), a pleiotropic cytokine, serves as a critical regulator of endothelial cell plasticity and repair processes. TGF-β1 exerts context-dependent effects on angiogenesis and tissue remodeling.^32^ Transient TGF-β1 activation promotes vascular stabilization via SMAD2/3—mediated quiescence signals, thereby supporting homeostasis. In contrast, chronic or excessive activation of TGF-β1 signaling promotes fibrosis and inflammation through alternative SMAD1/5 signaling or non-canonical pathways such as the mitogen-activated protein kinase (MAPK) pathway.^33–35^ Previous studies have demonstrated that TGF-β1 is highly expressed in BM microenvironment,^36,37^ with ECs displaying expression levels second only to megakaryocytes, and markedly higher than osteoblasts, perivascular stromal cells, and reticular cells.^38^ This unique spatial expression pattern suggests a specialized role of TGF-β1 in BM EC biology. Our recent investigations in the patients with AML, or AA indicated that aberrant upregulation of TGF-β1 signaling disrupts BM EC homeostasis and impairs their hematopoietic-supporting functions.^15,19^ Nevertheless, the causal role of TGF-β1 in initiating maladaptive BM EC repair and the downstream molecular cascades remain undefined. Importantly, pharmacologic interventions targeting TGF-β signaling have already entered the clinic. Luspatercept (a TGF-β ligand trap) has been shown to improve erythropoiesis in patients with MDS, and thalassemia.^39–41^ Its mechanism of action involves enhancing late-stage erythroid maturation. Beyond red cell recovery, emerging clinical trial data suggest that luspatercept may also improve white blood cell and platelet counts, raising the possibility that modulation of TGF-β signaling could benefit multilineage hematopoiesis.^42–45^ However, whether these therapeutic effects are mediated through restoring BM EC function, remains unexplored.

In the present study, we sought to address this critical gap by investigating the role of TGF-β1 signaling in BM EC maladaptive repair and its impact on hematopoietic regeneration. Using both in vitro models and murine systems with EC-specific overexpression of TGF-β receptor I (TGF-βRI), we examined how upregulated TGF-β1 signaling alters BM EC function and contributes to hematopoietic dysfunction. Multi-omics analyses were employed to uncover the molecular pathways driving these alterations. Furthermore, we conducted a prospective single-arm clinical study to evaluate the safety and efficacy of luspatercept in patients with poor hematopoietic reconstitution post allo-HSCT. By integrating mechanistic and translational approaches, our work aims to delineate the pathogenic role of TGF-β1 in BM EC maladaptation and to explore the potential of TGF-β1-targeted therapy as a strategy to promote multilineage hematopoietic recovery. Ultimately, these findings are expected to provide novel insights into BM vascular biology and inform the development of therapeutic interventions for hematologic disorders and transplantation-related complications.

## Results

### TGF-β1 activation impairs BM ECs and their hematopoietic-supporting ability

**Fig. 1:**
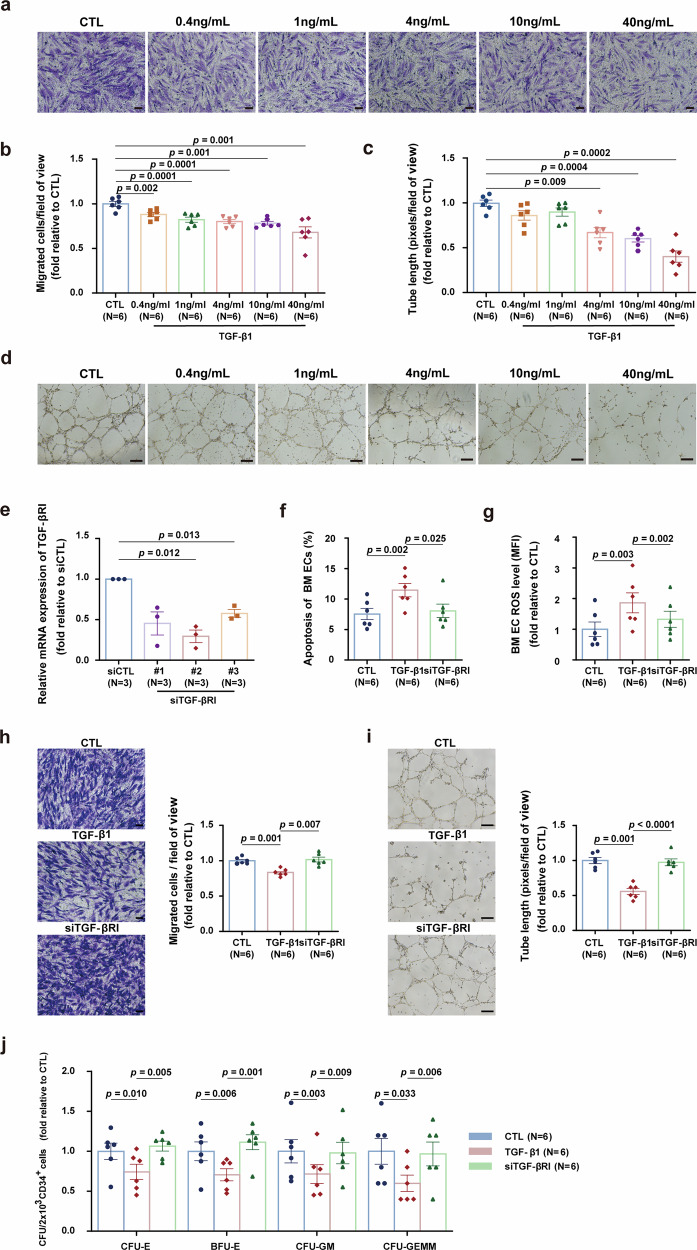
TGF-β1 activation induces bone marrow endothelial cell damage. Cultivated BM ECs from HDs subjects were incubated with varying concentrations of TGF-β1. The cells were collected for analyses of migration (**a**, **b**) and tube formation (**c**, **d**) (N = 6 per group, *n* = 3 per sample). **e** BM ECs cultivated from HDs were exposed to an siRNA aimed at TGF-βRI for 48 h, and the transfection efficiency was validated by qPCR (*N* = 3 per group, *n* = 1 per sample). **f** The percentages of apoptotic cultured BM ECs from HDs subjected to the indicated treatments were analyzed through flow cytometry (N = 6 per group, *n* = 1 per sample). **g** The ROS levels of the cultivated BM ECs of HDs subjected to the indicated treatments were analyzed through flow cytometry (N = 6 per group, *n* = 1 per sample). **h** Representative images (left; scale bars, 50 μm) and quantification (right) of migrated BM ECs in the indicated groups (N = 6 per group, *n* = 3 per sample). **i** Representative images (left; scale bars, 200 μm) and corresponding quantification (right) of tube formation (N = 6 per group, *n* = 3 per sample). **j** In the mentioned groups, BM ECs cultured from HDs were cocultured with CD34^+^ cells for a duration of 5 days to evaluate the colony-forming efficiency of CD34⁺ cells (CFU-E, BFU-E, CFU-GM, and CFU-GEMM; N = 6 per group, *n* = 3 per sample). N represents biological replicates; *n* represents technical replicates. The data are presented as the means ± SEMs. Statistical analyses were performed using paired t test. BM bone marrow, BFU-E burst-forming unit-erythroid, BM bone marrow, CFU colony-forming unit, CFU-E colony-forming unit-erythroid, CFU-GEMM colony-forming unit-granulocyte, erythroid, macrophage and megakaryocyte, CFU-GM colony-forming unit-granulocyte/macrophage, EC endothelial cells, HD healthy donor, ROS reactive oxygen species, SEM standard error of the mean

To investigate the impact of TGF-β1 pathway activation on BM ECs, normal BM ECs derived from HDs were treated with recombinant human TGF-β1 at different concentrations. Consistent with our previous results,^15^ TGF-β1 treatment impaired BM EC function in a concentration-dependent manner, as indicated by significantly inhibited cell migration and tube formation (Fig. 1a–d). Based on these results, an optimal concentration of 40 ng/mL was selected for the subsequent experiments.

To further confirm the role of the TGF-β1 pathway in BM ECs, we partially silenced TGF-βRI in BM ECs from HDs via siRNA transfection (Fig. 1e). Compared with cells treated with TGF-β1 alone (TGF-β1 group), those cells treated with both TGF-β1 and TGF-βRI siRNAs (siTGF-βRI group) exhibited significant reductions in apoptosis (Fig. 1f, 8.07 ± 1.10 vs. 11.47 ± 1.10, *p* = 0.025) and ROS levels (Fig. 1g, 1.33 ± 0.26-fold vs. 1.86 ± 0.33-fold, *p* = 0.002). Additionally, the silencing knockdown of TGF-βRI restored levels of cell migration (Fig. 1h, 1.02 ± 0.03-fold vs. 0.83 ± 0.02-fold, *p* = 0.007) and tube formation (Fig. 1i, 0.97 ± 0.05-fold vs. 0.56 ± 0.04-fold, *p* < 0.0001) in the TGF-β1-treated cells to levels comparable to those of the untreated control cells. Most importantly, TGF-βRI knockdown rescued the hematopoietic-supporting ability of BM ECs impaired by TGF-β1, as evidenced by the recovery of CFU-E (*p* = 0.005), BFU-E (*p* = 0.001), CFU-GM (*p* = 0.009), and CFU-GEMM (*p* = 0.006) values (Fig. 1j). Collectively, these results demonstrate that the activation of the TGF-β pathway induces BM EC damage and specifically compromises the hematopoietic-supporting ability of BM ECs. Moreover, our data demonstrate that the inhibition of the TGF-β pathway can ameliorate this damage and restore the compromised hematopoietic-supporting ability of BM ECs, thereby underscoring the therapeutic potential of the targeting of the TGF-β pathway for endothelial cell protection and hematopoietic regeneration.

### BM EC-specific TGF-βRI overexpression drives BM EC maladaptive repair and disrupts hematopoietic regeneration in mice

**Fig. 2:**
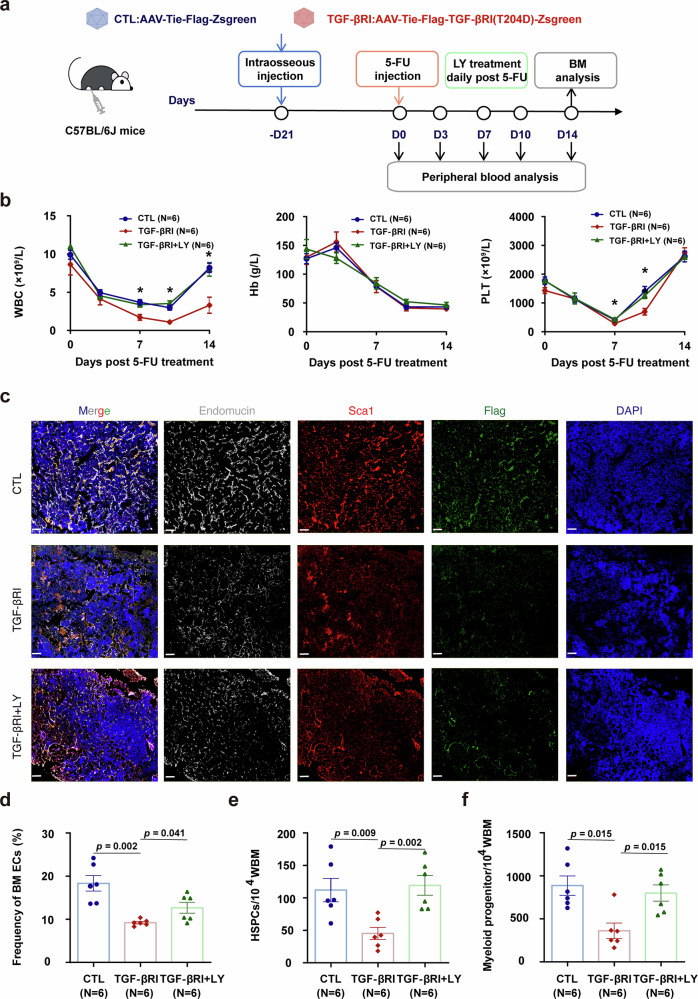
TGF-βRI overexpression in BM ECs exacerbates endothelial damage and delays hematopoietic recovery in mice. **a** Illustrative diagram of the study design involving mice with BM EC-specific overexpression of TGF-βRI. Adult female C57BL/6 J mice (8–10-weeks-old) were administered the recombinant AAV-V_EC_ via intraosseous injection. AAV-TGF-βRI mice (TGF-βRI) received AAV-V_EC_ encoding the TGF-βRI, Flag tag and ZsGreen genes under the regulation of a Tie promoter that is specific to ECs. TGF-βRI (T204D) indicates that the threonine at position 204 is replaced by aspartic acid, mimicking the persistent activation of TGF-βRI receptor. AAV-CTL mice (CTL), which were sex- and age-matched, received AAV-V_EC_ encoding only the Flag tag and ZsGreen genes under the regulation of the same Tie promoter. Cohorts of mice (TGF-βRI + LY) were subjected to treatment with 5-FU at a dose of 250 mg/kg via tail vein injection on day 0. LY2157299 (LY) or dimethyl sulfoxide (DMSO) was subsequently administered daily via intragastric gavage to the mice. On day 14, the mice were sacrificed. **b** Recovery kinetics of WBCs, Hb, and PLTs after the given treatments were analyzed (N = 6 per group, *n* = 3 per sample). **c** Representative fluorescent multiplex immunohistochemistry analysis of Endomucin^+^ (white) and SCA1^+^ (red) BM vessels and nuclei (blue) in femurs from the specified groups. Scale bars represent 100 μm. **d** The frequencies of CD31^+^VE^-^Cadherin^+^ECs within CD45^-^Ter119^-^ BM cells from mice subjected to the specified treatments were assessed through flow cytometry (N = 6 per group, *n* = 1 per sample). The frequencies of lineage-cKIT^+^SCA1^+^ HSPCs (**e**) and lineage^-^cKIT^+^SCA1^-^ MPs (**f**) in WBM cells were assessed via flow cytometry (N = 6 per group, *n* = 1 per sample). N represents biological replicates; *n* represents technical replicates. Data are expressed as mean ± SEM and analyzed by the Mann–Whitney U test. *p ≤ 0.05. EC endothelial cell, Hb hemoglobin, HSPC hematopoietic stem and progenitor cell, MP myeloid progenitor, PLT platelet, SEM standard error of the mean, WBM whole bone marrow, WBC white blood cell, 5-FU 5-fluorouracil

Based on our in vitro findings, we aimed to further investigate the role of the TGF-β1 pathway in BM ECs in vivo. We constructed a mutant vector of TGF-βRI, in which the threonine at position 204 was mutated to aspartic acid. This phosphomimetic mutation enables the receptor to mimic a persistently activated state (independent of the activities of TGF-β1 and TGF-βRII) by maintaining continuous phosphorylation. Using an Adeno-associated viruses (AAV) -mediated gene delivery system under the control of the EC-specific Tie promoter and via intra-BM injection, this mutant vector was specifically overexpressed in BM ECs in mice (Fig. 2a). Flow cytometry confirmed high BM EC transduction with undetectable targeting of HSCs, demonstrating both high efficiency and cell-type specificity (Supplementary Fig. 1).

Under steady state, mice with AAV-mediated TGF-β1 overexpression in ECs (AAV-TGF-βRI mice) exhibited comparable BM EC numbers and hematopoietic parameters compared to control mice (AAV-CTL mice). Furthermore, no significant differences were detected in the frequencies of hematopoietic stem and progenitor cells (HSPCs), lineage-committed cells, bone marrow vascular structures, or peripheral blood counts (Supplementary Fig. 2). To elucidate the role of the TGF-β1 pathway in compromised BM ECs during a state of stress hematopoiesis in vivo, AAV-CTL mice and AAV-TGF-βRI mice were subjected to 5-FU chemotherapy (Fig. 2a). At 14 days post 5-FU treatment, while transduction efficiency remained comparably high in BM ECs of both groups (Supplementary Fig. 3a), AAV-TGF-βRI mice exhibited significantly delayed peripheral blood (PB) recovery (Fig. 2b). Multiplex immunohistochemistry analysis revealed that, compared with AAV-CTL mice, AAV-TGF-βRI mice exhibited a significantly reduced number of BM ECs and impaired BM vessel structures (Fig. 2c, Supplementary Fig. 3b), which aligns with the results of the flow cytometry analysis (Fig. 2d, 9.23 ± 0.29 vs. 18.33 ± 1.81, *p* = 0.002). In conjunction with the observed maladaptive BM ECs in AAV-TGF-βRI mice, by day 14 following 5-FU treatment, compared to AAV-CTL mice, AAV-TGF-βRI mice exhibited a decrease in the percentages of HSPCs (Fig. 2e, 45.21 ± 9.37 vs.112.00 ± 18.02, *p* = 0.009) and myeloid progenitors (Fig. 2f, 360.90 ± 89.57 vs. 885.90 ± 112.70, *p* = 0.015), including granulocyte-macrophage progenitors, common myeloid progenitors, and megakaryocyte-erythrocyte progenitors in the BM of AAV-CTL mice. These findings highlight the pivotal role of the TGF-β1 pathway in mediating the maladaptive repair process of BM ECs, thereby resulting in BM EC damage and subsequent impairment of hematopoiesis.

### TGF-βRI inhibition restores BM ECs and accelerates hematopoietic recovery post-injury in vivo

To examine the effect of TGF-βRI inhibition on promoting BM EC regeneration in vivo, the TGF-βRI kinase inhibitor known as LY2157299 was administered to AAV-TGF-βRI mice after they were treated with 5-FU (Fig. 2a). PB analysis of AAV-TGF-βRI mice and AAV-TGF-βRI mice treated with LY2157299 revealed that TGF-βRI inhibition promoted PB cell recovery following 5-FU treatment (Fig. 2b). Compared with untreated AAV-TGF-βRI mice, AAV-TGF-βRI mice treated with LY2157299 demonstrated an increased number of BM ECs and improved BM vessel structures (Fig. 2c, d, 12.67 ± 1.27 vs. 9.23 ± 0.29, *p* = 0.041). Additionally, compared with untreated AAV-TGF-βRI mice, AAV-TGF-βRI mice receiving LY2157299 exhibited greater percentages of HSPCs (Fig. 2e, 119.30 ± 15.27 vs. 45.21 ± 9.37, *p* = 0.002) and myeloid progenitors (Fig. 2f, 799.60 ± 94.07 vs. 360.90 ± 89.57, *p* = 0.015) on day 14 post-5-FU treatment, thereby suggesting that BM ECs mediate hematopoietic protection. These data suggest that targeting the TGF-β1 pathway may represent a therapeutic strategy to promote the repair of BM ECs and expedite hematopoietic recovery.

### Transcriptomic shift from hematopoietic support to epithelial-mesenchymal transition mediators

**Fig. 3:**
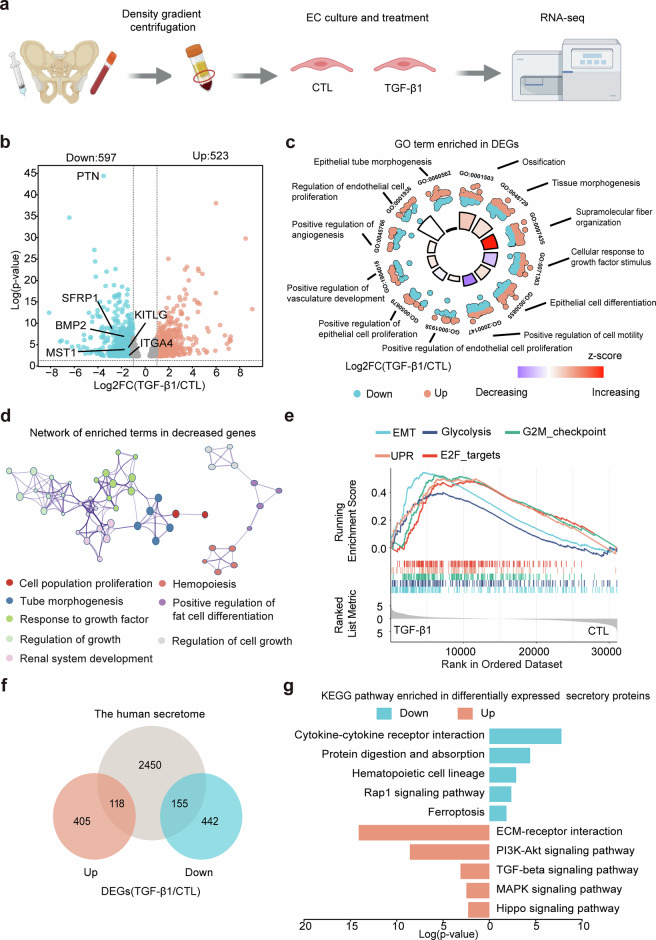
RNA-seq analysis revealed that TGF-β1 treatment induces a shift in the secretome of BM ECs. **a** Illustrative diagram of the RNA-seq analysis of BM ECs treated with or without TGF-β1. Certain elements of this figure were created with BioRender.com (www.biorender.com). **b** Distributions and quantifications of the genes in the indicated groups. The x-axis shows the log2 of the change in gene expression between the TGF-β1 group and the control group, whereas the y-axis represents -log₁₀(adjusted p value). **c** GO biological process analysis revealed the top significant GO terms in BM ECs treated with TGF-β1. The z-score is calculated as (number of up genes - number of down genes) divided by the square root of the total number of genes. **d** Network analysis revealed the enriched terms in BM ECs treated with TGF-β1. Each node within the network signifies an enriched term. Nodes are interconnected by edges when the associated terms display a similarity exceeding 0.3. The color of each node reflects its cluster ID, while the node’s size is determined by the number of genes included in the corresponding term. **e** The top 5 HALLMARK pathways enriched in BM ECs in the TGF-β1 group according to GSEA. **f** Venn diagram showing a significantly increased proportion of secretory proteins in BM ECs treated with TGF-β1. **g** KEGG analysis revealed the top significant pathways enriched with the differentially expressed genes encoding secretory proteins in BM ECs from the TGF-β1 group

**Fig. 4:**
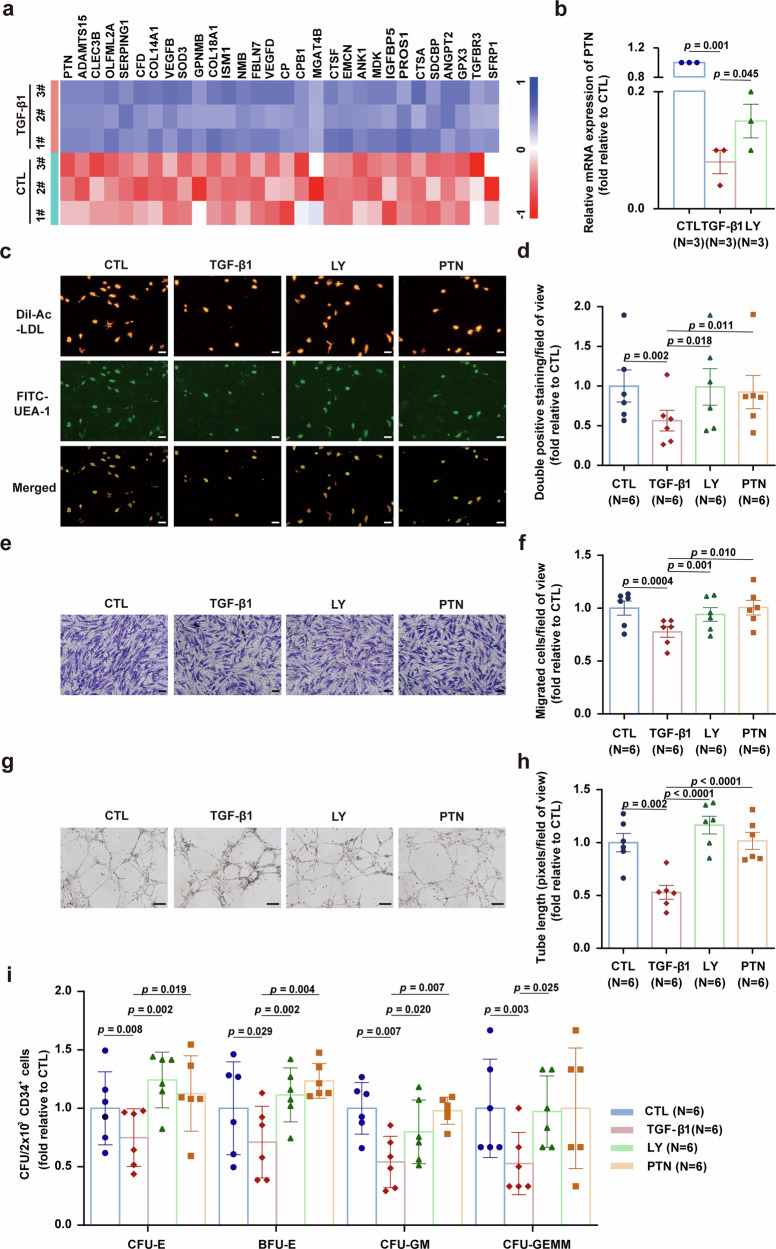
Either TGF-β1 pathway inhibition or HSC-active angiocrine factor pleiotrophin (PTN) treatment repairs maladaptive BM ECs in vitro. **a** Heatmap of the top 30 significantly downregulated genes encoding secretory proteins in BM ECs from the TGF-β1 group. **b** The mRNA level of PTN in BM ECs treated with TGF-β1 alone or in combination with LY2157299 (LY) was assessed via qPCR (N = 3 per group, *n* = 1 per sample). Representative images (scale bars, 50 μm) (**c**) and quantification (**d**) of double-positive BM ECs (yellow) co-stained with DiI-AcLDL (red) and FITC-UEA I (green) following the indicated treatments (N = 6 per group, *n* = 3 per sample). Quantification (**e**) and representative images (scale bars, 50 μm) (**f**) of migrated BM ECs following the indicated treatment (original magnification, ×10) (N = 6 per group, *n* = 3 per sample). Quantification (**g**) and representative images (scale bars, 200 μm) (**h**) of tube formation (pixels of tubes) of BM ECs after the indicated treatment (original magnification, 4 ×) (N = 6 per group, *n* = 3 per sample). **i** BM ECs cultured from HDs in the indicated groups were cocultured with CD34^+^ cells for a duration of 5 days to evaluate the colony-forming efficiency of CD34⁺ cells (CFU-E, BFU-E, CFU-GM, and CFU-GEMM; N = 6 per group, *n* = 3 per sample). N represents biological replicates; *n* represents technical replicates. The data are presented as the means ± SEMs. Statistical analyses were performed using paired t test. BFU-E burst-forming unit-erythroid, BM bone marrow, CFU colony-forming unit, CFU-E colony-forming unit-erythroid, CFU-GEMM colony-forming unit-granulocyte, erythroid, macrophage and megakaryocyte, CFU-GM colony-forming unit-granulocyte/macrophage, EC endothelial cell, SEM standard error of the mean

To mechanistically link the maladaptive EC repair phenotype observed both in vitro and in vivo to TGF-β1 signaling, we performed integrative transcriptomic and phosphoproteomic analyses (Fig. 3a). RNA-seq of TGF-β1-treated BM ECs revealed 523 upregulated and 597 downregulated genes ( | log2FC | >1, adj. *p* < 0.05) (Fig. 3b). Enrichment and protein-protein interaction analyses revealed that the genes downregulated by TGF-β1 were predominantly associated with critical endothelial functions, including the cellular response to growth factor stimulus, positive regulation of cell motility, angiogenesis, hemopoiesis and cell growth (Fig. 3c, d). Conversely, Gene set enrichment analysis (GSEA) of the hallmark gene set revealed significant upregulation of pathways linked to maladaptive remodeling, such as epithelial-mesenchymal transition (EMT), glycolysis, the G2M checkpoint, and the unfolded protein response (UPR) (Fig. 3e). Strikingly, among the downregulated genes, 26% of these genes encoded secretory proteins (Fig. 3f), which was greater than the genome-wide average of approximately 12%^37,46^ and included key hematopoietic regulators. Among these regulators, pleiotrophin (PTN), which is a key regulator of HSC regeneration, exhibited the most pronounced downregulation (Fig. 4a, 0.087-fold, *p* < 0.001). Similarly, critical niche factors, including *SFRP1* (0.15-fold), *BMP2* (0.28-fold), *MST1* (0.29-fold), *KITLG* (0.37-fold), *ITGA4* (0.45-fold), and *CSF-1* (0.59-fold, *p* ≤ 0.005 for all), were significantly downregulated. These genes collectively regulate HSPC maintenance, adhesion, and differentiation, with their coordinated suppression underscoring a systemic disruption of BM EC-mediated hematopoietic support. Enrichment analysis revealed that these downregulated secretory molecules were predominantly involved in cytokine-cytokine receptor interactions, hematopoietic cell lineages, and the Rap1 signaling pathway (Fig. 3g). Conversely, the upregulated secretome components were enriched in the ECM-receptor interaction, the PI3K-Akt signaling pathway and the MAPK signaling pathway, validated by qPCR (Fig. 3g, Supplementary Fig. 4). These molecular signatures align with our in vitro and in vivo findings, thereby collectively demonstrating that sustained TGF-β1 activation drives BM ECs toward a maladaptive repair process.

### Either TGF-β1 pathway inhibition or HSC-active angiocrine factor PTN treatment can repair dysfunctional BM ECs in vitro

To investigate the causal relationship between secretome dysregulation and endothelial damage, we focused on PTN, which is a prohematopoietic secretory factor that was identified as being the most significantly downregulated gene in TGF-β1-treated BM ECs via RNA-seq (Fig. 4a), with qPCR validation confirming its reduced expression after TGF-β1 treatment (Fig. 4b, 0.08 ± 0.02-fold, *p* = 0.001) and increased expression after LY treatment (Fig. 4b, 0.15 ± 0.03-fold, *p* = 0.045). To determine whether PTN deficiency mediates TGF-β1-induced maladaptive repair in BM ECs, we administered the TGF-βRI inhibitor LY2157299 (LY) or recombinant PTN to TGF-β1-treated cells. Both interventions quantitatively restored BM ECs, as evidenced by the recovery of DiI-AcLDL/FITC-UEA-1 dual-staining. Compared to TGF-β1-treated cells (0.56 ± 0.13-fold), LY treatment resulted in 0.99 ± 0.23-fold recovery (*p* = 0.018), while PTN supplementation achieved 0.92 ± 0.21-fold recovery (Fig. 4c, d, *p* = 0.011). Functional assays further demonstrated rescued migration. Relative to TGF-β1 treatment (0.78 ± 0.05-fold), LY treatment yielded 0.94 ± 0.06-fold recovery (*p* = 0.001), and PTN supplementation achieved 1.01 ± 0.07-fold recovery (Fig. 4e, f, *p* = 0.010). Similarly, tube formation assays showed LY treatment resulted in 1.17 ± 0.08-fold recovery (*p* < 0.0001) and PTN supplementation achieved 1.02 ± 0.08-fold recovery (*p* < 0.0001) versus TGF-β1-treated cells (Fig. 4g, h, 0.53 ± 0.07-fold). Critically, the hematopoietic-supporting ability was restored. Compared to TGF-β1 treatment, LY increased CFU-E colonies 1.24 ± 0.10-fold (*p* = 0.002), BFU-E colonies 1.12 ± 0.09-fold (*p* = 0.002), and CFU-GM/GEMM colonies 0.80 ± 0.11-fold/0.97 ± 0.12-fold (*p* = 0.020/*p* = 0.025). PTN supplementation increased CFU-E colonies 1.13 ± 0.13-fold (*p* = 0.019), BFU-E colonies 1.23 ± 0.06-fold (*p* = 0.004), and CFU-GM/GEMM colonies 0.98 ± 0.05-fold/1.00 ± 0.21-fold (Fig. 4i, *p* = 0.007/*p* = 0.095). These data collectively establish a link between PTN suppression and the maladaptive repair of TGF-β1-driven BM ECs, as well as highlight potential therapeutic interventions via TGF-β1 inhibition or PTN restoration.

### Time-course phosphoproteomics Identifies TGF-β1-driven dysregulation of VEGF/Notch signaling crosstalk and sustained p38α MAPK activation in EC maladaptation

**Fig. 5:**
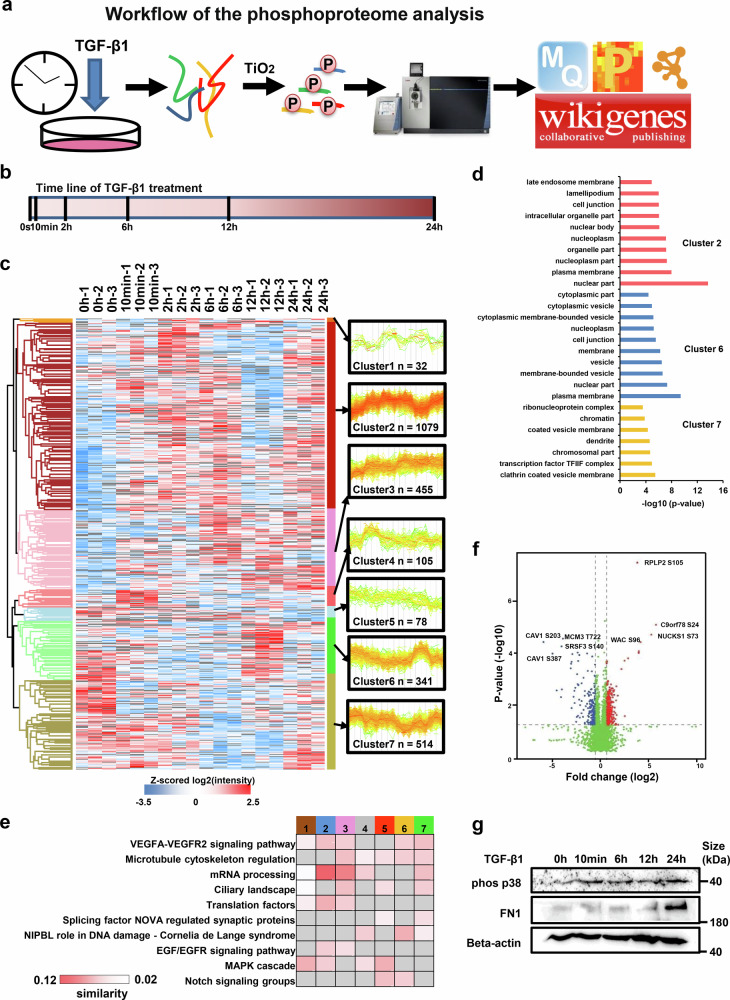
Temporal dissection of TGF-β1-regulated phosphorylation events. **a** Workflow of the phosphoproteome analysis. **b** Schematic overview of the time points used for TGF-β1 treatment (10 ng/ml). **c** Heatmap illustrating the outcomes of hierarchical clustering analysis based on z-scored intensities of phosphorylation (ANOVA test, *P* < 0.05). The phosphosites were clustered into seven categories according to their intensities. Seven clusters are also shown in the profile plot, with the numbers of included phosphosites indicated. **d** The bar graphs show the GOCC enriched in cluster 2, cluster 6 and cluster 7 of phosphoproteins. The analysis was performed using Perseus. **e** The similarity of the clustered pathways induced by TGF-β1. The profiles are color-coded according to their similarity from the respective cluster. The red scale represents the similarity score calculated via the WikiPathways analysis from the Cytoscape website, whereas gray represents missing values. **f** Volcano plots showing the indicated upregulated (red circles) and downregulated (blue circles) phosphosites after 12 h of TGF-β1 treatment. **g** HUVECs were treated for the indicated time periods with 10 ng/mL TGF-β1. The cell lysates were subjected to immunoblotting with anti-phospho-p38α (p-p38), anti-Fibronectin-1 (FN1) and anti-β-actin antibodies. The western blot analyses were performed in triplicate and representative images are shown

Time-course phosphoproteomic analysis (0–24 h of TGF-β1 stimulation) (Fig. 5a, b) mapped 16,981 phosphorylation sites with high reproducibility (Supplementary Fig. 5), which were clustered into seven distinct temporal patterns (Fig. 5c). Most phosphorylation changes occurred between 6 and 12 h (cluster 2, 6, and 7), suggesting a switch from transient to sustained cellular effects induced by TGF-β1. Enrichment of gene ontology (GO) terms indicates that the proteins containing downregulated phosphosites at 12-h in cluster 2 are enriched in nuclear-localized proteins and involved in metabolic/transcriptional regulation (Fig. 5d and Supplementary Figs. 6, 7). Cluster 2 exhibited progressive hyperphosphorylation except 12-h point, notably at MAPK14 (p38α T180/Y182) and AKT3 (S472/S476). These phosphosites were enriched in processes related to negative regulation of gene expression (Supplementary Fig. 7), suggesting sustained non-canonical TGF-β1 pathway activation. This is consistent with our previous findings that p38α phosphorylation induces functional abnormalities in BM ECs.^4,14^ The proteins containing upregulated phosphosites at 12-h in cluster 6 are involved in negative regulation of epithelial cell migration, and this is consistent with the observation that TGF-βRI silencing enhances cell migration (Fig. 1h). The GO term secretion is also enriched in cluster 6, indicative of proteins reside on cytoplasmic membrane-bound vesicles (Supplementary Fig. 7). In cluster 7, proteins involved in phosphatase activator activity are enriched (Supplementary Fig. 8), explaining the overall suppression of phosphorylation at this time point, particularly in clusters 2 and 7. The kinases involved in phosphorylation of the two clusters were also predicted according to their substrate motifs (Supplementary Fig. 9), and their activity were likely suppressed at the 12-h time point.

To further unveil the phosphorylation-mediated signaling pathways downstream of TGF-β1, the enrichment of pathways in different clusters was assayed using WikiPathways.^47^ Ten signaling pathways are enriched in more than two clusters, including VEGF, Notch, and cytoskeletal regulation as potential TGF-β1-responsive pathways (Fig. 5e). The proteins involved mRNA process are also enriched in four clusters (Supplementary Fig. 10), indicative of the regulatory role of phosphorylation in TGF-β1 stimulated mRNA processing. Notably, Notch pathway is enriched both in clusters 5 and 6, which implies that the phosphorylation of some proteins in the pathway was increased while the others was decreased at the 12-h time point. Specifically, the Notch pathway component NUMB (Fig. 5e, Supplementary Fig. 11), a known modulator of VEGF signaling that promotes angiogenesis through Notch antagonism and regulation of VEGF receptor endocytosis, exhibited decreased phosphorylation at its S227 residue following 12 h of observation. Cavin-1 (S203/S387) phosphorylation also decreased at 12 h, potentially disrupting caveolae-mediated endocytosis (Fig. 5f). Such a differential phosphorylation could affect VEGFR2 and VEGFR3 activation and regulate downstream signaling of VEGF (such as the PI3K/AKT and MAPK pathways) as well as the occurrence of EMT (Fig. 5g, Supplementary Fig. 12), because VEGFR2 and VEGFR3 are regulated in a highly differential manner by Notch.^48^ These findings delineate a TGF-β1-driven signaling nexus characterized by disordered VEGFR/Notch crosstalk and the subsequent activation of p38α, both of which combine to impair endothelial repair and niche function. This study offers a dynamic phosphoproteomic framework that unravels TGF-β1’s pathological role in vascular maladaptation.

### The TGF-β1 pathway is activated in maladaptive BM ECs from patients with PGF

**Fig. 6:**
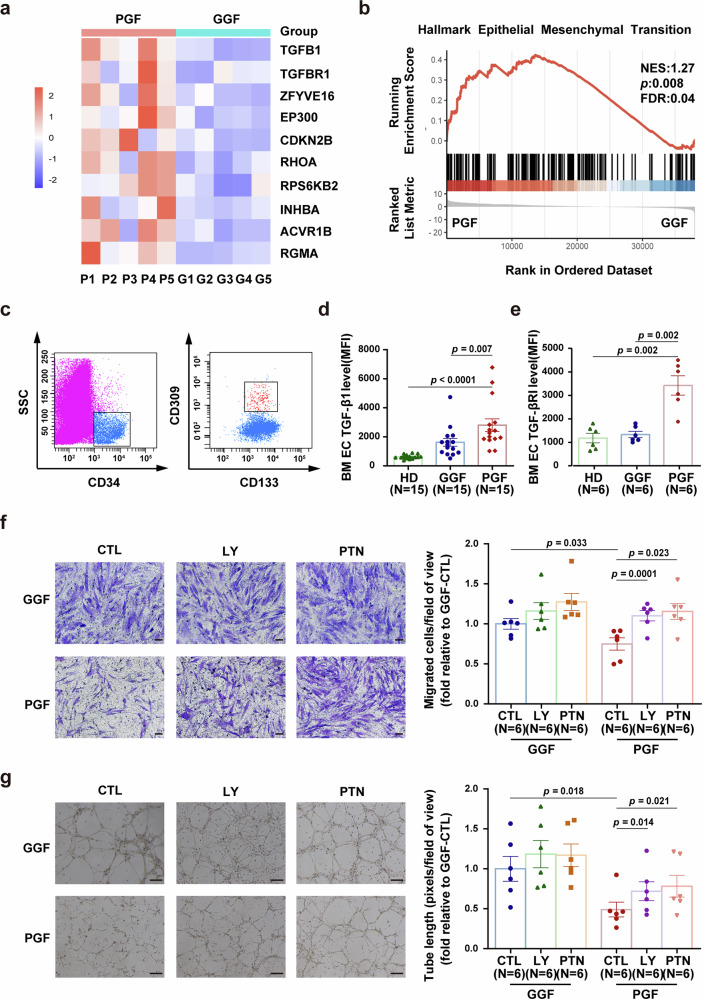
Hyperactive TGF-β1 pathway in BM ECs from PGF patients. **a** Heatmap of the selected TGF-β1 pathway genes in BM ECs isolated from PGF patients versus GGF patients. **b** GSEA showing enrichment of the epithelial–mesenchymal transition pathway in BM ECs from PGF patients. **c** To detect intracellular TGF-β1 and TGF-βRI levels in precultured BM mononuclear cells, BM ECs, which typically express CD309, CD34, and CD133, were first gated by using flow cytometry. Quantification of the intracellular levels of TGF-β1 (**d**, N = 15 per group, *n* = 1 per sample) and TGF-βRI (**e**, N = 6 per group, *n* = 1 per sample) levels in gated precultured BM ECs from PGF, GGF and HD was performed via flow cytometry, and the MFI was determined. The cultured BM ECs from the PGF or GGF patients were treated with LY2157299 (LY), PTN or control solvent. **f** Representative images (left panel; scale bars represent 50 μm) of migrated BM ECs following the indicated treatment (original magnification, ×10) and quantification (right panel) of migrated BM ECs (N = 6 per group, *n* = 3 per sample). **g** Representative images (left panel; scale bars represent 200 μm) and quantification (right panel) of tube formation of BM ECs after the indicated treatment (N = 6 per group, *n* = 3 per sample) (original magnification, ×4). N represents biological replicates; *n* represents technical replicates. The data are presented as the means ± SEMs. Statistical analyses were performed using the Mann–Whitney U test, unpaired t test and paired t test. BMMNC bone marrow mononuclear cells, EC endothelial cell, GGF good graft function, HD healthy donor, MFI mean fluorescence intensity, PGF poor graft function, PTN pleiotrophin, SEM standard error of the mean

Based on the aforementioned in vitro, murine, and mechanistic results, we aimed to explore the role of TGF-β1 signaling in patients with PGF posttransplantation. BM ECs from PGF patients (PGF-ECs) and their paired GGF patients (GGF-ECs) were isolated by fluorescence-activated cell sorting and analyzed by RNA sequencing.^27^ Re-analysis of these transcriptomic data revealed significant upregulation of TGF-β1, TGF-βRI, and downstream effectors in PGF-ECs compared with GGF-ECs (Fig. 6a). Consistently, GSEA demonstrated marked enrichment of the TGF-β-driven EMT pathway in PGF-ECs (Fig. 6b), in line with our in vitro multi-omics data from TGF-β1-stimulated ECs. To further validate these observations, a prospective nested case-control study was conducted to compare the expression of BM TGF-β1, and TGF-βRI in PGF-ECs and GGF-ECs. The representative BM EC phenotype was characterized as CD34^+^CD309^+^CD133^+^ via flow cytometry (Fig. 6c). The intracellular levels of BM TGF-β1 (Fig. 6d, 2803 ± 439 vs. 1625 ± 269, *p* = 0.007), and TGF-βRI (Fig. 6e, 3423 ± 411 vs. 1335 ± 135, *p* = 0.002) in PGF-ECs were significantly greater than those in GGF-ECs. These results imply that the TGF-β pathway was activated in BM PGF-ECs.

### TGF-β1 inhibition can restore the function of maladaptive BM ECs from PGF patients in vitro

Moreover, the TGF-β1 pathway inhibitor LY2157299 reinstated the migratory capacity of BM ECs in vitro (Fig. 6f, 1.10 ± 0.06-fold vs. 0.75 ± 0.08-fold, *p* = 0.0001) and promoted tube formation (Fig. 6g, 0.72 ± 0.12-fold vs. 0.49 ± 0.09-fold, *p* = 0.014). These findings suggest that the TGF-β1 pathway could serve as a potential therapeutic target for PGF patients.

### A prospective clinical trial involving luspatercept demonstrates significant promotion of hematopoiesis recovery in post-HSCT patients

**Fig. 7:**
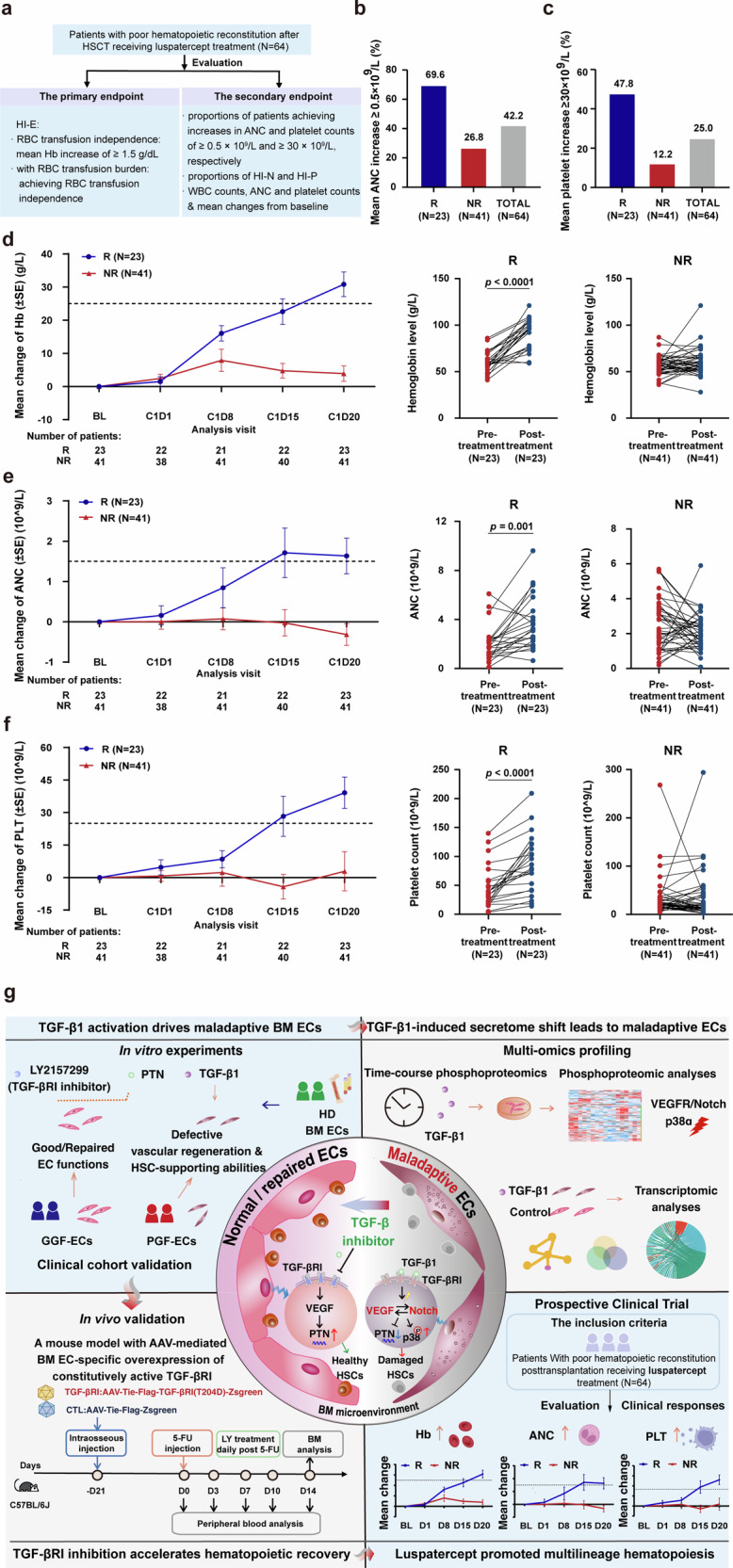
Clinical assessment of luspatercept in patients with poor hematopoietic reconstitution posttransplantation. **a** Study flowchart. Achievement of improved ANC (**b**) and PLT (**c**) at C1D20. **d-f** The baseline values were defined as the levels of Hb, ANC, and PLT before the first dose of luspatercept. Mean changes from baseline in the PB, including the levels of Hb (**d**), ANC (**e**), and PLT (**f**). With regard to the primary endpoint, the left panels show the mean change levels of Hb, ANC, and PLT over time between the R group and the NR group. The middle and right panels show the levels of Hb, ANC, and PLT before and after luspatercept treatment in the R group and the NR group. Paired t test was performed to compare the changes before and after treatment. Analysis visits are shown according to cycle (C) and day (D). The bars indicate the standard error. **g** Schematic illustration of TGF-β1-triggered maladaptive BM endothelium impeding hematopoietic recovery. Briefly, TGF-β1 drives BM EC maladaptation, thereby altering the secretome and impairing vascular repair and hematopoietic support. The inhibition of TGF-β1 reverses maladaptive ECs and enhances multilineage hematopoiesis recovery in post-HSCT patients. BL baseline, Hb hemoglobin, HSCT hematopoietic stem cell transplantation, RBC red blood cell, Hb hemoglobin, HI-E hematological improvement-erythroid, HI-P hematological improvement-platelet, HI-N hematological improvement-neutrophil, WBC white blood cell, ANC absolute neutrophil count, PLT platelet, R responder, NR non-responder

To assess the effect of a TGF-β ligand trap on hematopoietic recovery post-HSCT, a prospective clinical trial was performed in patients with poor hematopoietic reconstitution posttransplantation. The response rate of hematological improvement-erythroid (HI-E) was 35.9% (23 of 64 patients) (Fig. 7a). Notably, a mean absolute increase in neutrophils of ≥0.5 × 10^9^/L was observed in 16 patients (69.6%) from the responder (R) group and 11 (26.8%) patients from the non-responder (NR) group, respectively (Fig. 7b). Among these patients, the neutrophils of 4 and 6 patients were < 1 × 10^9^/L pretreatment, and 4/4 (100.0%) and 5/6 (83.3%) of the previously mentioned patients achieved hematological improvement-neutrophil (HI-N) in the R and NR groups, respectively. Besides, a mean increase in the platelet count of ≥ 30 × 10^9^/L was observed in 11 patients (47.8%) from the R group and 5 (12.2%) patients from the NR group, respectively (Fig. 7c). Among these patients, the platelet counts of 20 and 38 patients were < 100 × 10^9^/L before treatment, and 8/20 (40.0%) and 5/38 (13.2%) of the previously mentioned patients achieved HI-N in the R and NR groups, respectively. In addition, 6 patients achieved HI-N or HI-P, and 2 patients achieved both HI-N and hematological improvement-platelet (HI-P) in the NR group. Overall, the response rate of HI-N, HI-P or HI-E was 48.4% (31 of 64) in patients with poor hematopoietic reconstitution posttransplantation.

On day 20 after the first administration of luspatercept, the hemoglobin (Hb) concentrations, absolute neutrophil counts (ANCs), platelet counts, and white blood cell (WBC) counts were significantly elevated in patients achieving an HI-E response (N = 23) after treatment compared to before treatment (Fig. 7d–f, Supplementary Fig. 13a, b). In comparison, NR patients showed no substantial enhancement after treatment when assessed against their baseline levels (Fig. 7d–f, Supplementary Fig. 13c).

Our results indicated that luspatercept significantly promoted multilineage hematopoiesis recovery in post-HSCT patients who achieved an HI-E response without severe adverse events.

## Discussion

BM vascular microenvironment, centered around ECs, constitutes an indispensable unit for the maintenance, proliferation, differentiation of HSCs. This highly specialized microenvironment supports hematopoiesis not only through physical architectural support, but also via a complex network of angiocrine signals that finely regulate HSC fate decisions.^1–12^ However, BM ECs are particularly vulnerable to injury resulting from common clinical interventions such as chemotherapy and radiation, as well as pathological stresses such as chronic inflammation, hypoxia, and oxidative damage.^4–7,14–20^ While transient insults can often be resolved through adaptive repair pathways, persistent or recurrent activation of stress signaling cascades precipitates a transition to maladaptive repair. This aberrant repair state is characterized by fibrotic transcriptional reprogramming, loss of EC identity and functionality, and significantly diminished hematopoietic supportive capacity,^21,22^ ultimately contributing to serious clinical complications such as PGF following allo-HSCT.^4–7,17,23,24^ A central aim of the current study was to identify the molecular determinant that governs the switch from adaptive to maladaptive EC repair. Our findings provide compelling evidence that persistent activation of the TGF-β1 pathway acts as a master regulator of this transition. Using both in vitro models and an in vivo murine system with AAV-mediated EC-specific overexpression of a constitutively active TGF-βRI, we established a direct causal link between sustained TGF-β1 signaling, BM EC dysfunction, impaired vascular regeneration, and defective hematopoietic recovery. These results revealed that TGF-β1 hyperactivation is sufficient to drive ECs away from their pro-regenerative phenotype and toward a maladaptive state. Importantly, this dysfunction was not only limited to structural endothelial abnormalities but was also associated with functional collapse of hematopoietic support. These results highlight TGF-β1 as a pivotal driver of maladaptive EC remodeling in the BM microenvironment (Fig. 7g), mechanistically connecting chronic stress signaling with impaired hematopoietic reconstitution after transplantation.

Our results significantly extend current understanding of TGF-β1’s multifaceted role in tissue homeostasis and diseases. While TGF-β1 is widely recognized as a master regulator of fibrosis and EMT in solid organs,^32^ its function in BM vasculature has been less well defined. This study bridges an important knowledge gap by mechanistically linking sustained TGF-β1 activity to BM EC dysfunction. Our multi-omics approach elucidated the key mechanisms driving this process. Through a multi-omics approach, we delineated the molecular pathways underpinning this maladaptation. Transcriptomic profiling revealed a profound TGF-β1-driven secretome shift. Key hematopoietic-supportive factors—including PTN, KITLG, and BMP2—were suppressed, while mediators of EMT and fibrosis were upregulated. This rewiring of EC identity effectively converts ECs from a pro-hematopoietic, regenerative state into a dysfunctional, fibrotic one. Complementing these observations, our phosphoproteomic analysis uncovered that chronic TGF-β1 stimulation dysregulates VEGFR/Notch crosstalk, a signaling axis fundamental to angiogenic sprouting and vessel maturation. Sustained disruption of this crosstalk resulted in hyperphosphorylation and prolonged activation of p38α MAPK, a stress-response kinase and known driver of EMT. These results align with emerging evidences that the TGF-β, VEGF, and Notch pathways engage in dynamic crosstalk,^49–54^ and they expand the conceptual framework by identifying p38α as a downstream integrator of maladaptive endothelial signaling. Together, these data mechanistically link persistent TGF-β1 hyperactivation to vascular niche dysfunction, establishing TGF-β1 as a central orchestrator of maladaptive BM microenvironment remodeling.

The therapeutic implications of these insights are considerable. Pharmacological inhibition of TGF-βRI with LY2157299 in our murine model reversed the maladaptive phenotype, restoring peripheral blood counts, improving vascular architecture, and increasing the numbers of BM ECs and HSPCs. These results align with our previous observations in clinical models of AML and AA, wherein TGF-β1 inhibition rescued BM EC function and restored normal hematopoiesis.^15,19^ Collectively, these preclinical data underscore the therapeutic potential of targeting TGF-β1 signaling to rehabilitate the BM vascular niche. Importantly, our translational studies provide additional support for this therapeutic paradigm. Although current TGF-β1-targeting agents, such as luspatercept (a novel fusion protein that traps TGF-β ligands), are primarily used to enhance late-stage erythroid maturation in MDS and thalassemia,^39–41^ our clinical trial data underscore their diverse translational relevance. Specifically, luspatercept treatment in post-HSCT patients improved not only erythropoiesis but also panhematopoietic recovery. While larger, prospective randomized trials are necessary to confirm these benefits, our preliminary clinical findings suggest an expanded therapeutic paradigm: using luspatercept or other TGF-β1 inhibitors to promote trilineage hematopoiesis recovery in patients post-allo-HSCT by functionally rehabilitating the BM ECs. It is important to note that VEGF exhibits context-dependent effects on the hematopoietic-supporting ability of BM ECs,^15,19^ likely attributable to its crosstalk with TGF-β signaling.^55^ Consequently, the variable clinical efficacy of luspatercept among patients may be influenced by differing VEGF levels within the BM microenvironment, suggesting a potential role for biomarker-driven patient stratification in future applications.

Although our results support a central role for TGF-β1 in BM EC maladaptation, several limitations must be acknowledged. The BM microenvironment is a highly complex ecosystem comprising not only ECs but also mesenchymal stromal cells (MSCs), osteoblasts, adipocytes, and neural cells. Although our work demonstrates that EC-specific maladaptation is sufficient to impair hematopoiesis, it is probable that TGF-β1 signaling concurrently affects other BM niche constituents. MSCs, in particular, are known responders to TGF-β1 and to support hematopoiesis.^56,57^ Future studies employing cell-specific conditional knockout models will be essential to dissect the individual contributions of TGF-β1 signaling in each BM niche component and to fully understand the systemic effects of its inhibition.

In conclusion, our study elucidates a previously unrecognized mechanism whereby persistent TGF-β1 signaling orchestrates maladaptive repair in BM ECs. By reprogramming the endothelial secretome toward EMT mediators and disrupting VEGF–Notch signaling crosstalk, chronic TGF-β1 activation impairs vascular regeneration and undermines hematopoietic support, thereby contributing to PGF and related disorders. Moving beyond mechanistic insight, our preclinical data and clinical data collectively indicate pharmacological inhibition of the TGF-β1 pathway as a viable and pathogenesis-oriented therapeutic strategy. Targeting the dysfunctional BM microenvironment with agents such as TGF-β1 inhibitors holds considerable promise for restoring multilineage hematopoiesis and improving outcomes in patients following allo-HSCT, with potential implications for treating other BM failure conditions. Together, these findings establish a strong rationale for the clinical development of “niche-directed” therapies and open promising avenues for both mechanistic investigation and translational innovation in regenerative hematology.

## Materials and methods

### Patients and controls

#### Definitions

Poor graft function (PGF)^4,5,7,17,58^ is characterized by hypo/aplastic BM with at least two cytopenias persisting beyond 28 days after HSCT, including an absolute neutrophil count (ANC) below 0.5×10⁹/L, platelets under 20×10⁹/L, or hemoglobin less than 70 g/L. Additionally, PGF necessitates transfusions or G-CSF with full donor chimerism. Good graft function (GGF) is characterized by an ANC exceeding 0.5×10⁹/L, platelets over 20×10⁹/L, and hemoglobin above 70 g/L without transfusions after day +28. The details of the transplantation procedures have been previously documented.^6,59–62^

In the prospective nested case-control study, fifteen PGF patients were enrolled. A matched GGF patient was randomly selected for each PGF patient from the same allo-HSCT cohort at Peking University Institute of Hematology. The matching criteria encompassed pre-HSCT chemotherapy cycles count, age at HSCT, disease status at HSCT, and the timing of BM EC analysis post-HSCT, following “risk-set sampling”^63^ methodology. No notable differences were found in acute graft-versus-host disease (aGVHD), CD34^+^ cell dose, or cytomegalovirus (CMV) history (Table S1). The age-matched healthy donors (HDs, aged 20–55 years) were enrolled as the healthy control group.

#### Design of a prospective single-arm study

The study assessed the efficacy and safety of luspatercept in promoting normal hematopoiesis recovery in patients with poor hematopoietic reconstitution after allo-HSCT. The trial was registered at www.clinicaltrials.gov (NCT05629260). The inclusion criteria were patients aged 18–60 years with poor hematopoietic reconstitution post-HSCT, receiving luspatercept treatment,^41,64,65^ and adhering to the study and follow-up protocols. Patients with uncontrolled active infections, severe organ injury, or hypersensitivity to luspatercept were excluded.

The data cutoff for the clinical analysis was 20 days after the first administration of luspatercept. The primary endpoint, HI-E, was defined as the achievement of red blood cell (RBC) transfusion independence (TI) for patients previously dependent on transfusions, or a mean Hb increase ≥1.5 g/dL for RBC-TI patients at the first administration.^66^ In subsequent analyses, patients meeting the primary end point were categorized as R, while others were NR.^40^ Secondary endpoints included proportions of patients who achieved absolute increases in neutrophil ≥0.5 × 10^9^/L and platelet counts ≥30 × 10^9^/L, respectively; proportions of patients who achieved HI-N and HI-P counts; measurements of absolute neutrophil, platelet and white blood cell (WBC) counts; and mean changes from baseline. HI-N is characterized by a pretreatment neutrophil count of less than 1 × 10^9^/L and an absolute neutrophil increase of ≥ 0.5 × 10^9^/L following treatment. HI-P is characterized by an increase in platelet count of ≥ 30 × 10^9^/L in patients with pretreatment levels below 100 × 10^9^/L.^64,66^

#### Isolation, cultivation, and characterization of primary BM ECs

Primary BM mononuclear cells (BMMNCs) from HDs, PGF patients, and GGF patients were stained with the typical EC markers known as CD45⁻/CD34⁺/CD133⁺/VEGFR2⁺, anti-TGF-β1 (Abcam), and TGF-βRI (Abcam) for flow cytometry via the BD LSRFortessa analyzer (Becton Dickinson Biosciences) as previously reported.^4–7,14–17,19^

BMMNCs from HDs, PGF patients, and GGF patients were isolated with lymphocyte isolation solution via density gradient centrifugation. The cells were subsequently cultured in fibronectin-coated 24-well plates using EGM-2-MV-SingleQuots medium with 10% fetal bovine serum. Details of the functional assays such as DiI-AcLDL uptake and FITC-UEA-I binding assay, apoptosis, migration, reactive oxygen species (ROS), tube formation, and colony-forming unit (CFU) assays are provided in the Supplementary Methods.

#### Gene modulation and drug treatment in vitro

For gene silencing, small interfering RNA (siRNA) against TGF-βRI was synthesized (target sequence:5’-CTTACAGCATTGCGGATTA-3’) (RiboBio) was transfected into BM ECs cultivated from HDs for 7 days. Transfections were carried out using Lipofectamine 3000 (Invitrogen), with cells pre-seeded at a density of 2 × 10^5^ per 60-mm dish 24 h prior to transfection. The efficiency was assessed 48 h after transfection by measuring TGF-βRI expression levels using qPCR.

BM ECs were treated with TGF-β1 (40 ng/mL, PeproTech), LY2157299 (100 μM, MedChemExpress), and Pleiotrophin (PTN, 100 ng/mL, PeproTech) for 24 h. TGF-β1 was washed out before LY or PTN was added, and these reagents were subsequently removed prior to functional and co-culture experiments. After treatment, BM ECs were detached by using trypsin containing 0.25% EDTA (Gibco).

#### Establishment of murine models for myelosuppression and BM EC-specific TGF-βRI overexpression

A murine myelosuppression model^12,17,67–69^ was constructed via the administration of 5-FU (250 mg/kg) to C57BL/6 J female mice (8-10 weeks old). To establish a BM EC-specific TGF-βRI overexpression model, we used a constitutively active mutant of TGF-β receptor I (T204D) as previously described,^70^ in which the threonine residue at position 204 was substituted with aspartic acid to mimic persistent receptor activation via sustained phosphorylation (independent of the interaction between the TGF-β1 ligand and TGF-βRII). This genetic construct was delivered via an AAV-mediated gene delivery system^17,71^ (a recombinant AAV-V_EC_ optimized for EC transduction), regulated by the EC-specific Tie promoter. Intraosseous administration was performed to achieve localized BM EC transduction, following the established protocols for hematopoietic niche targeting.^17,70,72–75^ To assess the impact of BM EC-specific TGF-βRI overexpression and its pharmacological inhibition on BM EC damage, mice were injected with 5-FU (250 mg/kg) via tail vein on day 0, followed by daily oral gavage of LY2157299 (100 mg/kg, Selleck) beginning on the same day and continuing throughout the experiment. The kinetics of the PB were analyzed at days 3, 7, 10, and 14. Myeloid progenitors (lineage^-^cKIT^+^SCA1^-^), hematopoietic stem and progenitor cells (HSPCs, lineage^-^cKIT^+^SCA1^+^), HSCs (lineage^-^cKIT^+^SCA1^+^CD150^+^CD48^-^), B cells, T cells, myeloid cells, and ECs (CD45^-^Ter119^-^CD31^+^VE^-^Cadherin^+^)^15–17,19,76^ were utilized. Hematoxylin and eosin (H&E) staining and multiplex immunohistochemistry for the EC marker Endomucin (Emcn) were conducted. Stem cell antigen-1 (SCA1) was used in all of the mice.

All of the mouse experiments were approved by the Ethics Committee of Peking University People’s Hospital.

#### Phosphopeptide enrichment

Human umbilical vein endothelial cells (HUVECs) were lysed using a 4% SDC buffer and subsequently boiled for 10 min at 100 °C. Proteins were subjected to overnight digestion with trypsin at a 1:50 (w/w) ratio at 37 °C. Phosphopeptides were enriched using titanium dioxide beads (GL Sciences) following the protocol described by Humphrey et al.^77^

#### Nanoflow liquid chromatography (nLC) tandem mass spectrometry (MS)

The phosphoproteome was analyzed by an Orbitrap Fusion Lumos mass spectrometer (Thermo Fisher Scientific) coupled online to an Easy-nLC 1000. The samples were separated on a C18 column using a 110 min nonlinear gradient. For the MS scans, the mass range and resolution were set at 300–1500 m/z and 120,000, respectively. The automatic gain control (AGC) target was configured to 1×10^6^. For the MS2 scans, MS/MS spectra were obtained in a 3 s cycle between master scans; additionally, the fragment ions were detected in Orbitrap with a resolution of 30,000, and the AGC target was set at 5 × 10^4^.

### Data analysis

The database search was performed in the MaxQuant environment (version 2.3.1.0).^78^ The UniProt human proteome database containing 20,244 entries was used for the database search. Modifications of different factors and structures were established, such as the oxidation of methionine, protein N-terminal acetylation, and phosphorylation of serine, threonine, and tyrosine residues. Other parameters were set to the default values.

## Supplementary information

Clinical study protocol

Supplementary Materials

## Data Availability

The RNA-seq data associated with accession number HRA012063 have been released. The proteomics dataset (ProtemoeXchange ID: PXD065074; Project ID: IPX0012302000) will be made publicly available upon publication, following submission of the required journal metadata to the iProX repository.
