# Supplementary material for: TGF-β1-triggered maladaptive bone marrow endothelium impedes hematopoietic recovery
Source: Signal Transduct Target Ther. 2025 Oct 7;10:332. doi: 10.1038/s41392-025-02429-y (PMC12500950; doi:10.1038/s41392-025-02429-y)
Supplement: Supplementary file 1 — Clinical study protocol [file 41392_2025_2429_MOESM1_ESM.docx]

**CLINICAL STUDY PROTOCOL**

**Protocol Title: Luspatercept for promoting hematopoietic recovery in patients with poor hematopoietic reconstitution after allogeneic hematopoietic stem cell transplantation——a prospective single-arm clinical study**

**Study Drug:** **Luspatercept**

**Indication: poor hematopoietic reconstitution after allogeneic hematopoietic stem cell transplantation**

**Applicant Institution:** **Peking University People’s Hospital**

**Principal institution: Peking University People’s Hospital**

**Version: 1.0**

**Date: 20220905**

**Remark: the primary version of this protocol was in Chinese. We have translated it into English.**

**INVESTIGATOR'S STATEMENT**

I have received and completely reviewed the following protocol (Protocol Version 1.0, dated 05 September 2022), including all appendices:

As Principal Investigator, I understand and agree to conduct this clinical study as described and will comply with the ethical and regulatory considerations delineated herein.

**Study Title**

Luspatercept for promoting hematopoietic recovery in patients with poor hematopoietic reconstitution after allogeneic hematopoietic stem cell transplantation——a prospective single-arm clinical study

**Principal Investigator Signature and Contact Information**

**Principal Investigator (print) Xiao-Jun Huang**

**Principal Investigator (signature)
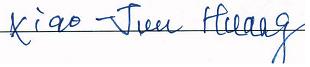
**

**Date of Signature 2022-09-05**

**Institution/Affiliation Peking University People’s Hospital**

**City, Province, Country Beijing, China**

# Study Synopsis

| **Study title** | Luspatercept for promoting hematopoietic recovery in patients with poor hematopoietic reconstitution after allogeneic hematopoietic stem cell transplantation——a prospective single-arm clinical study |
| --- | --- |
| **Indication** | Poor hematopoietic reconstitution after allogeneic hematopoietic stem cell transplantation |
| **Study applicant** | Peking University People’s Hospital |
| **Study center** | Peking University People’s Hospital |
| **Number of subjects planned** | Approximately 64 subjects |
| **Study duration** | Estimated to be 3 year |
| **Objectives** | **Primary objective:**  To evaluate the efficacy and safety of luspatercept for promoting hematopoiesis recovery in patients with poor hematopoietic reconstitution after allogeneic hematopoietic stem cell transplantation (allo-HSCT).  **Exploratory objectives:**  To evaluate the influence of luspatercept in the bone marrow microenvironment among patients with poor hematopoietic reconstitution after allo-HSCT. |
| **Study design** | This is a prospective single-arm clinical study to evaluate luspatercept for promoting hematopoietic recovery in patients with poor hematopoietic reconstitution after allo-HSCT.  Approximately 64 subjects will receive luspatercept treatment.  The dose of luspatercept ranged from 0.7 to 1.4 mg/kg subcutaneously on the basis of routine supportive therapy.  The complete blood count will be monitored every 7 days within 20 days from the beginning of luspatercept treatment.  Adverse events were evaluated following the National Cancer Institute Common Toxicity Criteria version 5.0. |
| **Inclusion criteria** | Subjects eligible for enrolment in this study must meet all of the following criteria:   1. Poor hematopoietic reconstitution after HSCT receiving luspatercept treatment 2. Aged 18-60 years 3. Comply with the study and follow-up procedures |
| **Exclusion criteria** | Subjects meeting any of the following criteria are ineligible for this study:   1. Severe organ injury 2. Uncontrolled active infections 3. Hypersensitivity to luspatercept |
| **Study treatment** | **Luspatercept treatment**  Poor hematopoietic reconstitution after HSCT will be enrolled, and luspatercept will be administered subcutaneously. The complete blood count (CBC) will be monitored every 7 days.  The primary endpoint was defined as hematologic improvement-erythroid (HI-E), which included the achievement of red blood cell transfusion independence (RBC-TI) for patients requiring RBC transfusions before administration, as well as a mean Hb increase ≥ 1.5 g/dL for RBC-TI patients at the first administration. The secondary endpoints included proportions of patients who achieved absolute increases in neutrophil ≥ 0.5 × 10^9^/L and platelet counts ≥ 30 × 10^9^/L, respectively; proportions of patients who achieved HI-neutrophil (HI-N) and HI-platelet (HI-P) counts; measurements of white blood cell (WBC), absolute neutrophil and platelet counts; and mean changes from baseline. |
| **Sample size determination** | The number of patients eligible for luspatercept treatment was estimated based on the 20-day hematologic improvement-erythroid (HI-E) reported by Vanstraelen et al.[^1^](#_ENREF_1_1) The present study was planned to detect a 20-day ORR of 28% in patients receiving luspatercept treatment from the reference rate of 14%, controlling for type I and II error rates at 5 and 20%, respectively. Considering an expulsion rate of 10%, a total of 64 patients were planned to be enrolled. |
| **Statistical analysis** | Statistical analyses will be performed based on the intent-to-treat (ITT) population.  **Primary Efficacy Analysis:**  Primary Outcome Measure:  1. Hematologic improvement-erythroid (HI-E)   1. For patients requiring RBC transfusions before administration: the achievement of red blood cell transfusion independence (RBC-TI) 2. For RBC-TI patients at the first administration: mean Hb increase ≥ 1.5 g/dL   **Secondary Efficacy Analysis:**  1. Hematologic improvement-leukocyte  1) Proportions of patients who achieved absolute increases in neutrophil ≥ 0.5 × 10^9^/L;  2) Proportions of patients who achieved HI-neutrophil (HI-N):  HI-N was defined as a neutrophil count of < 1 × 10^9^/L at pretreatment and the achievement of an absolute neutrophil increase of ≥ 0.5 × 10^9^/L after treatment;  3) Measurements of white blood cell (WBC), absolute neutrophil and mean changes from baseline.  2. Hematologic improvement-platelet  1) Proportions of patients who achieved absolute increases in platelet counts ≥ 30 × 10^9^/L;  2) Proportions of patients who achieved HI-platelet (HI-P):  HI-P was defined as a platelet increase of ≥ 30 × 10^9^/L among patients with pretreatment levels < 100 × 10^9^/L;  3) Measurements of platelet counts and mean changes from baseline.  **Safety Analyses:**  Safety and tolerability will be assessed by the incidence and severity of AEs and changes from baseline of all relevant parameters, including laboratory test values, physical examination findings, vital signs, and ECOG performance scores. With the exception of haematologic AEs, all AEs will be graded according to the National Cancer Institute Common Toxicity Criteria version 5.0. All subjects will be monitored for AEs within 20 days post-treatment. |
|  |  |

**TABLE OF CONTENTS**

PAGE

[Study Synopsis 3](#_Toc170678164)

[1. Introduction 12](#_Toc170678165)

[2. Study objectives 12](#_Toc170678166)

[2.1 Primary Objective 12](#_Toc170678167)

[2.2 Secondary Objectives 13](#_Toc170678168)

[3. Study Design 13](#_Toc170678169)

[4. Subject Selection Criteria 14](#_Toc170678170)

[4.1 Subject Selection Criteria 14](#_Toc170678171)

[4.1.1 Number of Subjects 14](#_Toc170678172)

[4.1.2 Inclusion Criteria 14](#_Toc170678173)

[4.1.3 Exclusion Criteria 14](#_Toc170678174)

[4.2. Withdrawal Criteria 14](#_Toc170678175)

[5. Study Procedures 15](#_Toc170678176)

[5.1 Screening 15](#_Toc170678177)

[5.2 Treatment Allocation and Blinding 15](#_Toc170678178)

[5.3 Study Treatment 16](#_Toc170678179)

[5.4 Follow-up 16](#_Toc170678180)

[6. Efficacy Assessments 16](#_Toc170678181)

[6.1 Definitions 16](#_Toc170678182)

[6.2 Primary Efficacy Endpoint 16](#_Toc170678183)

[6.3 Secondary Efficacy Endpoints 17](#_Toc170678184)

[6.4 Schedule and Methods of Efficacy Assessments 17](#_Toc170678185)

[7. Safety Evaluation 17](#_Toc170678186)

[7.1 Medical History 18](#_Toc170678187)

[7.2 Vital Signs and Physical Examination 18](#_Toc170678188)

[7.3 Clinical Symptoms 18](#_Toc170678189)

[7.4 Clinical Laboratory Evaluations 18](#_Toc170678190)

[8 Adverse Events and Serious Adverse Events (SAEs) 19](#_Toc170678191)

[8.1. Definitions 19](#_Toc170678192)

[8.1.1 Adverse Events 19](#_Toc170678193)

[8.1.2 Serious Adverse Events 20](#_Toc170678194)

[8.2 Assessment of Severity 20](#_Toc170678195)

[8.3 Assessment of Causality 21](#_Toc170678196)

[8.4 Recording and Reporting of AEs and SAEs 22](#_Toc170678197)

[9. Rules of Withdrawal 22](#_Toc170678198)

[9.1. Subjects Withdraw from the Study 22](#_Toc170678199)

[9.2. Premature Termination of the Study 23](#_Toc170678200)

[10. Rules of Follow-Up 23](#_Toc170678201)

[10.1 Follow-up Period 23](#_Toc170678202)

[10.2 Visit Scheduling 23](#_Toc170678203)

[10.3 Contents 23](#_Toc170678204)

[11. Data Analysis and Statistical Considerations 23](#_Toc170678205)

[11.1 Study Design Considerations 23](#_Toc170678206)

[11.1.1 Sample Size Assumptions 24](#_Toc170678207)

[11.1.2 Primary Efficacy Endpoint 24](#_Toc170678208)

[11.1.3 Secondary Efficacy Endpoints 24](#_Toc170678209)

[11.2 Data Analysis Considerations 25](#_Toc170678210)

[11.2.1 Analysis Population 25](#_Toc170678211)

[11.2.2 Analysis Plan 25](#_Toc170678212)

[11.2.2.1 Baseline Data 25](#_Toc170678213)

[11.2.2.2 Analysis of Efficacy 25](#_Toc170678214)

[11.2.2.3 Analysis of Safety 25](#_Toc170678215)

[12. Materials for the Study 26](#_Toc170678216)

[13. Ethical Considerations 26](#_Toc170678217)

[13.1 Responsibility of Investigators 26](#_Toc170678218)

[13.2 Informed Consent Process 26](#_Toc170678219)

[13.3 Good Clinical Practice 27](#_Toc170678220)

[13.4 Protection of Subjects’ Personal Data 27](#_Toc170678221)

[14. Administrative Requirements 27](#_Toc170678222)

[15. References 27](#_Toc170678223)

[16 Appendices 29](#_Toc170678224)

[16.1 Appendix 1 29](#_Toc170678225)

**Abbreviations**

| ADL | Activities of daily living |
| --- | --- |
| AEs | Adverse events |
| Allo-HSCT | Allogeneic hematopoietic stem cell transplantation |
| ANC | Neutrophil |
| AST | Aspartate aminotransferase |
| BM | Bone marrow |
| CRFs | Case report forms |
| ECs | Endothelial cells |
| ECOG | Eastern Cooperative Oncology Group |
| GCP | Good clinical practice |
| Hb | hemoglobin |
| HI-E | Hematologic improvement-erythroid |
| HI-P | Hematologic improvement-platelet |
| HI-N | Hematologic improvement-neutrophil |
| HSCs | Hematopoietic stem cells |
| MDS | Myelodysplastic neoplasm |
| PLT | Platelets |
| RBC-TI | Red blood cell transfusion independence |
| SAEs | Serious adverse events |
| TGF-β1 | Transforming growth factor-beta 1 |
| WBC | White blood cell |

# 1. Introduction

Transforming growth factor-beta 1 (TGF-β1), which is a pleiotropic cytokine, demonstrates context-dependent roles in angiogenesis and tissue repair. Transient TGF-β1 activation promotes vascular stabilization via SMAD2/3-mediated quiescence signals, whereas chronic activation drives fibrosis and inflammation through SMAD1/5 or non-canonical pathways (such as the MAPK pathway).[^2-4^](#_ENREF_1_2) Previous studies have demonstrated that TGF-β1 is highly expressed in bone marrow (BM).[^5^](#_ENREF_1_5) Among the various cells constituting the BM microenvironment, endothelial cells (ECs) exhibit a relatively high expression level of TGF-β1, surpassed only by megakaryocytes and significantly higher than that in osteoblasts, perivascular cells, and reticular cells.[^6^](#_ENREF_1_6) This unique spatial expression pattern provides the foundation for our investigation into the role of TGF-β1 signalling in BM ECs.

Luspatercept (a TGF-β ligand trap) has been reported to improve erythropoiesis in patients with myelodysplastic neoplasm (MDS) and thalassemia,[^7-11^](#_ENREF_1_7) and some clinical trials have suggested potential benefits for white blood cell and platelet counts.[^12-14^](#_ENREF_1_12) However, the ability of luspatercept to promote the recovery of multilineage hematopoiesis by reversing BM EC maladaptation remains unexplored.

This is a prospective single-arm clinical study to evaluate the efficacy and safety of luspatercept for promoting hematopoiesis recovery in patients with poor hematopoietic reconstitution after allo-HSCT.

# 2. Study objectives

## 2.1 Primary Objective

To evaluate the efficacy and safety of luspatercept for promoting hematopoiesis recovery in patients with poor hematopoietic reconstitution after allogeneic hematopoietic stem cell transplantation (allo-HSCT).

## 2.2 Secondary Objectives

To evaluate the influence of luspatercept in the bone marrow microenvironment among patients with poor hematopoietic reconstitution after allo-HSCT.

# 3. Study Design

This is a prospective single-arm clinical study to evaluate luspatercept for promoting hematopoietic recovery in patients with poor hematopoietic reconstitution after allo-HSCT.

Patients with poor hematopoietic reconstitution after HSCT receiving luspatercept treatment will be enrolled, and the dose of luspatercept ranged from 0.7 to 1.4 mg/kg subcutaneously on the basis of routine supportive therapy. The complete blood count will be monitored every 7 days within 20 days from the beginning of luspatercept treatment. The primary endpoint was defined as hematologic improvement-erythroid (HI-E), which included the achievement of red blood cell transfusion independence (RBC-TI) for patients requiring RBC transfusions before administration, as well as a mean Hb increase ≥ 1.5 g/dL for RBC-TI patients at the first administration. The secondary endpoints included proportions of patients who achieved absolute increases in neutrophil ≥ 0.5 × 10^9^/L and platelet counts ≥ 30 × 10^9^/L, respectively; proportions of patients who achieved HI-neutrophil (HI-N) and HI-platelet (HI-P) counts; measurements of white blood cell (WBC), absolute neutrophil and platelet counts; and mean changes from baseline.

All the subjects will be followed for safety and tolerability within 20 days post-treatment. With the exception of haematologic adverse effects (AEs), all AEs will be graded according to the National Cancer Institute Common Toxicity Criteria version 5.0.

AEs= adverse effects; allo-HSCT= allogeneic hematopoietic stem cell transplantation; BM= bone marrow; EC= endothelial cell; HI-E= hematologic improvement-erythroid; HI-N= hematologic improvement-neutrophil; HI-P= hematologic improvement-platelet; MDS= myelodysplastic neoplasm; RBC-TI= red blood cell transfusion independence; TGF-β1= transforming growth factor-beta 1; WBC= white blood cell.

# 4. Subject Selection Criteria

## 4.1 Subject Selection Criteria

### 4.1.1 Number of Subjects

Approximately 64 patients with poor hematopoietic reconstitution after allo-HSCT will receive luspatercept.

### 4.1.2 Inclusion Criteria

Subjects eligible for enrolment in this study must meet all of the following criteria:

1. Poor hematopoietic reconstitution after HSCT receiving luspatercept treatment
2. Aged 18-60 years
3. Comply with the study and follow-up procedures

### 4.1.3 Exclusion Criteria

Subjects meeting any of the following criteria will be ineligible for this study:

1. Severe organ injury
2. Uncontrolled active infections
3. Hypersensitivity to luspatercept

## 4.2. Withdrawal Criteria

Subjects will be free to withdraw consent and discontinue participation in the study at any time and without prejudice towards future treatment. A subject's participation in the study may be discontinued at any time at the discretion of the investigator. Justifiable reasons for a subject to be withdrawn from the study include the following:

1. Inability to fully comply with the study protocol

2. Unacceptable toxicity

3. Best interest of the subject based upon the investigator’s discretion

4. At the request of the study subject at any time and for any reason

Subjects will be followed up unless informed consent is withdrawn. The reason for withdrawal from study participation and the date must be documented in the case report form (CRF). The investigator must complete the last visit, including vital signs, physical examination findings, laboratory tests, disease status and AE assessment, all of which must be documented in the CRF.

# 5. Study Procedures

## 5.1 Screening

Subjects with poor hematopoietic reconstitution after allo-HSCT will be screened for eligibility. Medical history evaluation, vital signs, physical examination, ECOG performance status, blood and urine sampling for laboratory tests, electrocardiogram, chest imaging examination and BM assessment will be performed to determine study eligibility, all of which must be performed ≤3 days prior to enrolment.

## 5.2 Treatment Allocation and Blinding

This is an open-label study. Neither the subjects nor the investigators will be blinded to the treatment. Upon completion of all the required screening assessments, eligible subjects will receive luspatercept treatment.

## 5.3 Study Treatment

After enrolment, patients in the study group (luspatercept arm) will be scheduled for luspatercept treatment. If the patients meet the inclusion criteria, they will receive luspatercept (0.7 to 1.4 mg/kg) subcutaneously. In cases of grade 3 or worse AEs (not including haematologic recovery), dose modifications, including dose reductions or interruptions, will be permitted at the discretion of the physician.

## 5.4 Follow-up

From the beginning of the first administration, the complete blood count will be monitored every 7 days within 20 days.

All the subjects will be followed for safety and tolerability within 20 days after luspatercept treatment. With the exception of haematologic AEs, all AEs will be graded according to the National Cancer Institute Common Toxicity Criteria version 5.0.

# 6. Efficacy Assessments

## 6.1 Definitions

- HI-E is defined as hematologic improvement-erythroid;
- HI-N is defined as a neutrophil count of < 1 × 10^9^/L at pretreatment and the achievement of an absolute neutrophil increase of ≥ 0.5 × 10^9^/L after treatment;
- HI-P is defined as a platelet increase of ≥ 30 × 10^9^/L among patients with pretreatment levels < 100 × 10^9^/L.

## 6.2 Primary Efficacy Endpoint

1. Hematologic improvement-erythroid (HI-E)

1. For patients requiring RBC transfusions before administration: the achievement of red blood cell transfusion independence (RBC-TI)
2. For RBC-TI patients at the first administration: mean Hb increase ≥ 1.5 g/dL

## 6.3 Secondary Efficacy Endpoints

1. Hematologic improvement-leukocyte

1) Proportions of patients who achieved absolute increases in neutrophil ≥ 0.5 × 10^9^/L;

2) Proportions of patients who achieved HI-neutrophil (HI-N);

3) Measurements of white blood cell (WBC), absolute neutrophil and mean changes from baseline.

2. Hematologic improvement-platelet

1) Proportions of patients who achieved absolute increases in platelet counts ≥ 30 × 10^9^/L;

2) Proportions of patients who achieved HI-platelet (HI-P);

3) Measurements of platelet counts and mean changes from baseline.

## 6.4 Schedule and Methods of Efficacy Assessments

The patient's peripheral blood will be drawn to assess the recovery of WBC, ANC, Hb and platelets after the administration of luspatercept.

# 7. Safety Evaluation

All the subjects enrolled in the study will be evaluated for safety and tolerability. Safety and tolerability will be assessed by the incidence and severity of AEs and changes from baseline of all relevant parameters, including laboratory test values, physical examination findings, vital signs, and ECOG performance scores. With the exception of haematologic AEs, all AEs will be graded according to the National Cancer Institute Common Toxicity Criteria version 5.0.

### 7.1 Medical History

Each subject's medical history will be obtained at screening. Information on any prior or existing medical conditions will be recorded on the appropriate CRF.

### 7.2 Vital Signs and Physical Examination

Vital signs and physical examination results must be documented before randomization, once a week after enrolment. The next 8 steps must be performed:

⚫ Physical examination

⚫ Heart rate

⚫ Blood pressure

⚫ Body temperature

⚫ Rate of respiration

⚫ Body weight

⚫ ECOG performance status

⚫ Signs of infection

### 7.3 Clinical Symptoms

During the study, the patients' clinical symptoms must be documented. The clinical symptoms may be associated with the administration of luspatercept reported previously.

### 7.4 Clinical Laboratory Evaluations

Before initiation of the study, the monitors will document the normal range of each test in every involved laboratory. During the study, the following steps will be performed:

⚫ Routine blood: white cell counts, neutrophil cell counts, hemoglobin, and platelet counts

⚫ Hepatic function: total bilirubin (both direct bilirubin and indirect bilirubin must be documented when total bilirubin is elevated), ALT, AST, lactic dehydrogenase, alkaline phosphatase, albumin and total protein

- Renal function: serum creatinine, urea nitrogen and uric acid
- Other biochemical indicators: amylase and lipase
- Electrolytes: sodium, potassium, calcium and magnesium
- Coagulation function: prothrombin time, prothrombin time-international normalized ratio, activated partial thromboplastin time and fibrinogen
- Urinalysis: protein, glucose and erythrocyte
- Electrocardiogram
- Chest imaging examination

## 8 Adverse Events and Serious Adverse Events (SAEs)

The investigator is responsible for detecting, documenting and reporting events that meet the definition of an AE or SAE.

### 8.1. Definitions

## 8.1.1 Adverse Events

An AE is any untowards medical occurrence in a subject of a clinical investigation, which does not necessarily have a causal relationship with the medicinal product. Therefore, an AE can be any unfavourable and unintended sign, including an abnormal laboratory finding, symptom, or disease (new or exacerbated), whether or not it is considered to be related to the product. This definition includes any newly occurring event or previous condition that has increased in severity or frequency since the administration of the product. However, haematologic recovery or death due to persistent nonremission should not be recorded as AEs.

## 8.1.2 Serious Adverse Events

A serious adverse event is any untowards medical occurrence that, at any dose:

- - Results in death
  - Is life-threatening
  - Requires hospitalization or prolongation of existing hospitalization—i.e., the AE requires at least a 24-hour inpatient hospitalization or prolongs a hospitalization beyond the expected length of stay.

Hospitalization or prolongation of existing hospitalization for social reasons will not be reported as an SAE.

- - Results in disability/incapacity
  - Results in congenital anomalies/birth defects
  - Important medical events

Medical or scientific judgement should be exercised in deciding whether SAE reporting is appropriate in other situations. An important medical event is an event that may not result in death, be life-threatening, or require hospitalization but is clearly of major clinical importance. The AE may jeopardize the subject or require intervention to prevent a serious outcome.

## 8.2 Assessment of Severity

With the exception of hematologic AEs, all AEs will be graded according to the National Cancer Institute Common Toxicity Criteria version 5.0. When the National Cancer Institute Common Toxicity Criteria version 5.0 criteria do not apply, severity will be defined according to the following criteria:

| **Severity** | **Description** |
| --- | --- |
| Grade 1- Mild | Asymptomatic or mild symptoms; clinical or diagnostic observations only; intervention not indicated |
| Grade 2- Moderate | Minimal, local or noninvasive intervention indicated; limiting age-appropriate instrumental activities of daily living (ADL) |
| Grade 3- Severe | Medically significant but not immediately life threatening; hospitalization or prolongation of hospitalization indicated; disabling; limiting self-case ADL |
| Grade 4- Life-threatening | Life-threatening consequences; urgent intervention indicated |
| Grade 5- Death | Death |

## 8.3 Assessment of Causality

Investigators must determine the relationship between each AE or SAE and the study treatment. The relationship between an AE or SAE and the study treatment will be defined according to the following criteria:

- - Definite: There is a clear temporal relationship to the study treatment, with no other possible cause
  - Possible: A temporal relationship to the study treatment is not clear, and alternative aetiologies are possible
  - Not related: There is no temporal relationship to the study treatment, and/or there is evidence of an alternative cause, such as a concurrent medication or illness

## 8.4 Recording and Reporting of AEs and SAEs

All AEs and SAEs must be recorded in the appropriate CRF, regardless of whether they are causally related to the study treatment. Each SAE must be reported promptly on the Serious Adverse Event Report Form and submitted to the Independent Ethics Committee within 24 hours by the investigator. The information recorded on the Serious Adverse Event Report Form will include at least the following: subject number, identity of the event, study drug name and dose, investigator's assessment of the event's severity and relationship to study treatment, and investigator's name and signature. When examining original medical records, clinical monitors must collect and verify detailed information on AEs and SAEs. All AEs and SAEs should be followed up until they are resolved.

# 9. Rules of Withdrawal

## 9.1. Subjects Withdraw from the Study

Subjects can withdraw from the study at any time for any reason without affecting the investigator’s right to treat the disease. Given the interest of the subjects, the investigator has the right to request that the subjects withdraw from the study for any reason, including concomitant disease, AEs or treatment failure. The core group of clinical studies reserves the right to request that subjects withdraw from the study for deviation(s) from the protocol, administrative reasons, or other effective or ethical reasons.

The last assessment for subjects must be performed and documented in the CRF regardless of the time and reason for withdrawal. The reason for withdrawal from study participation must be documented in the CRF. All documents related to subjects should be completed. Despite withdrawal from the study, those subjects should be followed up and documented about their diseases until withdrawal of informed consent.

For subjects who withdraw from the study due to concomitant diseases or AEs, the details must be documented in the CRF with other appropriate and valuable data attached.

## 9.2. Premature Termination of the Study

Reasons for premature termination of the study include external events, repetition of SAEs, an increasing incidence of treatment-related death and slow study enrolment. All the subjects will be informed of the premature termination of the study via written consent. Any subject who decides to discontinue participation in the study must report to the principal investigator.

# 10. Rules of Follow-Up

## 10.1 Follow-up Period

Starting from the signing of the informed consent form.

## 10.2 Visit Scheduling

Every 7 days until the study is completed.

## 10.3 Contents

The contents of every follow-up visit will include subject’s complaints, vital signs, physical examination, clinical symptoms and clinical laboratory evaluations (haematology, serum chemistry, urinalysis, electrocardiogram, chest imaging examination, and BM assessment). All of the results must be documented in the original medical records.

# 11. Data Analysis and Statistical Considerations

## 11.1 Study Design Considerations

This is a prospective single-arm clinical study to evaluate luspatercept for promoting hematopoietic recovery in patients with poor hematopoietic reconstitution after allo-HSCT. The dose of luspatercept ranged from 0.7 to 1.4 mg/kg subcutaneously on the basis of routine supportive therapy. In patients with symptoms of drug allergies, such as nausea, vomiting, rash, and bronchospasm, or in patients with disease relapse, the drug will be discontinued immediately.

### 11.1.1 Sample Size Assumptions

The number of patients eligible for luspatercept treatment was estimated based on the 20-day hematologic improvement-erythroid (HI-E) reported by Vanstraelen et al.[^1^](#_ENREF_1_1) The present study was planned to detect a 20-day ORR of 28% in patients receiving luspatercept treatment from the reference rate of 14%, controlling for type I and II error rates at 5 and 20%, respectively. Considering an expulsion rate of 10%, a total of 64 patients were planned to be enrolled.

### 11.1.2 Primary Efficacy Endpoint

The primary endpoint was defined as hematologic improvement-erythroid (HI-E), which included the achievement of red blood cell transfusion independence (RBC-TI) for patients requiring RBC transfusions before administration, as well as a mean Hb increase ≥ 1.5 g/dL for RBC-TI patients at the first administration.

### 11.1.3 Secondary Efficacy Endpoints

The secondary endpoints included proportions of patients who achieved absolute increases in neutrophil ≥ 0.5 × 10^9^/L and platelet counts ≥ 30 × 10^9^/L, respectively; proportions of patients who achieved HI-neutrophil (HI-N) and HI-platelet (HI-P) counts; measurements of white blood cell (WBC), absolute neutrophil and platelet counts; and mean changes from baseline.

## 11.2 Data Analysis Considerations

### 11.2.1 Analysis Population

Sixty-four patients with poor hematopoietic reconstitution after allo-HSCT at Peking University People’s Hospital will be enrolled in the luspatercept group.

### 11.2.2 Analysis Plan

### 11.2.2.1 Baseline Data

Baseline characteristics will be summarized and described in a frequency list.

### 11.2.2.2 Analysis of Efficacy

The definitions of the efficacy endpoints have been described in detail in the previous section.

The efficacy endpoints will be hematologic improvement-erythroid, hematologic improvement-leukocyte and hematologic improvement-platelet. GraphPad Prism 10 (GraphPad Software, Inc., La Jolla, CA) will be used for the statistical analyses. The Mann‒Whitney U test will be used for continuous variables, and paired t tests will be performed to analyse the matched or paired data. *P* values <0.05 will be considered to indicate statistical significance.

### 11.2.2.3 Analysis of Safety

Safety and tolerability will be assessed by the incidence and severity of AEs and changes from baseline of all relevant parameters, including laboratory test values, physical examination findings, vital signs, and ECOG performance scores. The definition of AEs has been detailed in previous section. With the exception of haematologic AEs, all AEs will be graded according to the National Cancer Institute Common Toxicity Criteria version 5.0. All subjects will be monitored for AEs within 20 days post-treatment. Categorical data will be summarized by the proportion of total subjects. Quantitative data will be described using arithmetic averages or medians for central tendencies and standard deviations or interquartile ranges for distribution ranges.

# 12. Materials for the Study

All materials that will be provided to the study sites and investigators are as follows:

- The study protocol
- Informed consent
- CRF

# 13. Ethical Considerations

## 13.1 Responsibility of Investigators

The investigators are responsible for guaranteeing the compliance of the clinical study with the protocol, the Chinese good clinical practice (GCP) guidelines and applicable laws and regulations.

## 13.2 Informed Consent Process

Prior to participation in the study, the subjects must be informed about the objectives, methods, possible benefits, potential risks and possible discomforts of the study by the investigators. They also should be informed that participation in the study would be voluntary, that they can withdraw from the study at any time, that there is no impact on the treatment of the disease whether they take part in the study, and that their privacy will be protected.

Subjects or their legally acceptable representatives should have enough time to read the informed consent form and raise queries. Written informed consent must be obtained from each subject or their legally acceptable representative.

## 13.3 Good Clinical Practice

This study will be conducted in accordance with the Declaration of Helsinki and the Chinese GCP. The study will be conducted only if it is approved by the ethical review committee of the principal study site. The investigators will guarantee that the study will be conducted in accordance with applicable laws and regulations and the scientific and ethical principles of the People’s Republic of China. If the protocol needs revision during the study, the revised version must be reapproved by the ethical review committee of the principal study site. If new data related to study treatment are discovered, the informed consent must be revised, and the revision must be reapproved by the ethical review committee of the principal study site and subjects.

## 13.4 Protection of Subjects’ Personal Data

The data collected in the study are limited in terms of efficacy and safety related to the study treatment. Data will be collected and used in accordance with applicable laws and regulations.

# 14. Administrative Requirements

Neither the investigator nor the applicant can revise the protocol without agreeing on the opposite side. All revisions of the protocol must be released by the applicant institution. To ensure the integrity, accuracy and reliability of the data, the relevant examination and treatment results must be documented in the original medical records and the CRF. Independent clinical monitoring will be performed regularly by a panel of qualified and experienced study investigators composed of haematologists who are blinded to the treatment assignments.

# 15. References

1 Vanstraelen, G. *et al.* Recombinant human erythropoietin therapy after allogeneic hematopoietic cell transplantation with a nonmyeloablative conditioning regimen: Low donor chimerism predicts for poor response. *Experimental Hematology* **34**, 841-850 (2006).

2 ten Dijke, P. & Arthur, H. M. Extracellular control of TGFbeta signalling in vascular development and disease. *Nat Rev Mol Cell Biol* **8**, 857-869 (2007).

3 Oh, S. P. *et al.* Activin receptor-like kinase 1 modulates transforming growth factor-beta 1 signaling in the regulation of angiogenesis. *Proc Natl Acad Sci U S A* **97**, 2626-2631 (2000).

4 Pardali, E. & ten Dijke, P. Transforming growth factor-beta signaling and tumor angiogenesis. *Front Biosci (Landmark Ed)* **14**, 4848-4861 (2009).

5 Uhlen, M. *et al.* Proteomics. Tissue-based map of the human proteome. *Science* **347**, 1260419 (2015).

6 Zhao, M. *et al.* Megakaryocytes maintain homeostatic quiescence and promote post-injury regeneration of hematopoietic stem cells. *Nat Med* **20**, 1321-1326 (2014).

7 Suragani, R. N. *et al.* Transforming growth factor-beta superfamily ligand trap ACE-536 corrects anemia by promoting late-stage erythropoiesis. *Nat Med* **20**, 408-414 (2014).

8 Zhou, L. *et al.* Inhibition of the TGF-beta receptor I kinase promotes hematopoiesis in MDS. *Blood* **112**, 3434-3443 (2008).

9 Platzbecker, U. *et al.* Luspatercept for the treatment of anaemia in patients with lower-risk myelodysplastic syndromes (PACE-MDS): a multicentre, open-label phase 2 dose-finding study with long-term extension study. *Lancet Oncol* **18**, 1338-1347 (2017).

10 Fenaux, P. *et al.* Luspatercept in Patients with Lower-Risk Myelodysplastic Syndromes. *N Engl J Med* **382**, 140-151 (2020).

11 Cappellini, M. D. *et al.* A Phase 3 Trial of Luspatercept in Patients with Transfusion-Dependent β-Thalassemia. *N Engl J Med* **382**, 1219-1231 (2020).

12 Cappellini, M. D., Marcon, A., Fattizzo, B. & Motta, I. Innovative Treatments for Rare Anemias. *HemaSphere* **5**, e576 (2021).

13 Suragani, R. N. *et al.* Transforming growth factor-β superfamily ligand trap ACE-536 corrects anemia by promoting late-stage erythropoiesis. *Nat Med* **20**, 408-414 (2014).

14 Garcia-Manero, G. *et al.* Hematologic Improvement-Neutrophil and -Platelet in the MEDALIST Trial: Multilineage Data from a Phase 3, Randomized, Double-Blind, Placebo-Controlled Study of Luspatercept to Treat Anemia in Patients with Very Low-, Low-, or Intermediate-Risk Myelodysplastic Syndromes (MDS) with Ring Sideroblasts (RS) Who Require Red Blood Cell (RBC) Transfusions. *Blood* **134**, 4243 (2019).

#

# 16 Appendices

## 16.1 Appendix 1

**ECOG Performance Status**

| **Grade** | **ECOG** |
| --- | --- |
| **0** | Fully active, able to carry on all predisease performance without restriction |
| **1** | Restricted in physically strenuous activities but ambulatory and able to carry out work of a light or sedentary nature, e.g., light house work or office work |
| **2** | Ambulatory and capable of all selfcare but unable to carry out any work activities; up and about more than 50% of waking hours |
| **3** | Capable of only limited selfcare; confined to bed or chair more than 50% of waking hours |
| **4** | Completely disabled; cannot carry out any selfcare; totally confined to bed or chair |
| **5** | Dead |

Oken MM, Creech RH, Tormey DC, Horton J, Davis TE, McFadden ET, Carbone PP. Toxicity And Response Criteria Of The Eastern Cooperative Oncology Group. Am J Clin Oncol. 1982; 5(6):649-655.
