# Supplementary material for: TGF-β1-triggered maladaptive bone marrow endothelium impedes hematopoietic recovery
Source: Signal Transduct Target Ther. 2025 Oct 7;10:332. doi: 10.1038/s41392-025-02429-y (PMC12500950; doi:10.1038/s41392-025-02429-y)
Supplement: Supplementary file 2 — Supplementary Materials [file 41392_2025_2429_MOESM2_ESM.docx]

Supplementary Materials for

TGF-β1-triggered maladaptive bone marrow endothelium impedes hematopoietic recovery

**Authors:** Zhong-Shi Lyu^1,2#^, Meng-Zhu Shen^1#^, Yuan-Ya Zhang^3#^, Hui Gao^3,4^, Mi Liang^1^, Yu-Hong Chen^1^, Zhen-Kun Wang^1^, Xin-Yan Zhang^1^, Dan-Dan Chen^1^, Yuan-Yuan Zhang^1^, Meng Lv^1^, Xiao-Do Mo^1^, Lan-Ping Xu^1^, Xiao-Hui Zhang^1^, Yu Wang^1^, Ying-Chun Wang^3,4^*, Yuan Kong^1^*, Xiao-Jun Huang^1,2^*

^#^These authors contributed equally.

*****These authors jointly supervised this work

Correspondence to: huangxiaojun@bjmu.edu.cn; successky@163.com; ycwang@genetics.ac.cn

**This PDF file includes:**

Materials and Methods

Figures. S1 to S13

Table S1

**Materials and Methods**

Cultivation, characterization and functional analyses of primary BM ECs

Bone marrow mononuclear cells (BMMNCs) from patients with poor graft function (PGF), good graft function (GGF), and healthy donors (HDs) were cultured in fibronectin-coated plates (Sigma) using EGM-2-MV medium (Lonza) supplemented with 10% fetal bovine serum (Gibco) at 37°C under 5% CO₂. After 7 days, adherent endothelial cells (ECs) were harvested using trypsin-EDTA (Gibco), counted via trypan blue exclusion (Solarbio), and characterized via flow cytometry (LSRFortessa, BD Biosciences) with CD34, CD45, VEGFR2 (CD309), and CD133 markers. Apoptosis levels were assessed via Annexin V/7-AAD staining followed by flow cytometry. For reactive oxygen species (ROS) measurement, cells were incubated with 10 μM DCFH-DA for 15 minutes, and fluorescence intensity was quantified.

Migration assays employed Transwell inserts, where 5×10⁴ cells in serum-free medium were seeded into upper chambers, while complete medium (10% FBS) was added to lower chambers; migrated cells were fixed, stained with crystal violet, and counted microscopically after 24 hours.

Tube formation was evaluated by plating 5×10⁴ cells on Matrigel-coated plates for 48 hours, with tubular networks imaged and analyzed using Image ProPlus. For functional characterization, ECs were incubated with DiI-AcLDL (10 μg/mL) for 4 hours, fixed, and stained with FITC-UEA-1 (10 μg/mL), with double-positive cells visualized by fluorescence microscopy.

CD34⁺ hematopoietic progenitor cells isolated from HDs via magnetic beads were co-cultured with treated ECs in StemSpan SFEM medium for 5 days, followed by colony-forming unit (CFU) assays in MethoCult H4434 for 14 days. CFU-E, BFU-E, CFU-GM, and CFU-GEMM colonies were enumerated by light microscopy, with triplicate replicates per condition.

Plasmid construction and adeno-associated virus (AAV) packaging

To create an AAV expression vector, we first constructed a shuttle plasmid carrying the T204D-mutated mTgfbr1 (NM_009370.4) sequence. This sequence was amplified and inserted into the BamHI- and PacI-digested pHBAAV-TIE-3flag-T2A-ZsGreen plasmid (HanHeng Biotechnology, Shanghai, Cat. No. AAV104) using seamless cloning. The ligation product was transformed into DH5α competent cells. After 12 - 16 h of incubation at 37°C, bacterial colonies were screened. Positive clones were identified by PCR and validated by sequencing. The verified plasmid was named pAAV-TIE-m- TGF-βR1/mut-Null-T2A-ZsGreen (pAAV-TGF-βR1/mut). For AAV production, pAAV-TGF-βR1/mut, the packaging plasmid pAAV-RC, and the helper plasmid pHelper (HanHeng Biotechnology) were co-transfected into AAV-293 cells at a mass ratio of 1:1:2. To enhance endothelial cell transduction, pAAV-RC was engineered with a mutant AAV2 Cap sequence for the VEC serotype. AAV-293 cells were cultured and harvested 72 h post-transfection to extract and purify recombinant AAV particles (overexpressing TGF-βR1). The viral genome titer was measured using SYBRGreen, yielding 1.4×10¹³ vg/mL for HBAAV2/VEC-Tie-m-TGF-βR1/mut-Null-ZsGreen.

Tie promoter sequence^1^ used in our study is as follows: CTTAAGACATGCAACTCGTCTACGGCTATACCACTCTGAACGCGCCCGATCTCGGAAGACATGCAACTCAAATGTAAATACAGTAGAATATTACTTAGGTAGAAACTCCTGGTGATTTTAAAAGATTGGAAAAGAATATGAGGAAGAGTTGAATAATGCAAATTCTAGTGTGTGTGCTACCGAAGTGAACACTTAATGCACAGTCTACAGACTAGGACATTTTATCGTGTGTTGTAAAATTGGGTAGAAACTTGTGTTTGTGAAAACTGAGCATTAAAACCTTACAGAGACCGTTTCTTGTTTACTTTTGAAAAAAAAAAGAGTCACGTGAGCCTCATTTTGTATTTGTGTGTGTGTGTGTGTGTGTGTCTCCCCTCCTCCCAGCGTGTGTGTGCTGGGAGGAGGGGAGACCCCAGAACAATGTCCTGCCTCCAAACCTTCTCAATAGGCGGAAGCCACTGGCTTCCTCCCTTTCCTGTCTCCCGTGCTCCAGCAATGCAGATGGAAGGGACCGAAGGGATGGGAGAGAGAGCCCAACCATCCCCAGATCTGTCCTTGTCACAACCTGCCTCCCACCTCTAATGCCCCCCCTTCCAGAGACTTCCAGGCCACACCCATCCCGGGCTTGTGGGGGCTGGACACGGGAGGACTACAGGCGACAACTCTTCCCACCCTCTCTCCCTGCCACCCCTCCTACCCTAACCATCATTTCCTCTTCCTCCCCAGCACCGAGGTGCACTGAGCTGGACAGGCTGAACACTCAGACCCACAGCAACTGACCCCGGGCCC

DNA sequence encoding the TGF-βR1 mutant with Threonine at position 204 mutated to Aspartic Acid（p.T204D (ACC to GAC)）ATGGAGGCGGCGGCCGCTGCTCCACGTCGTCCGCAGCTCCTCATCGTGTTGGTGGCGGCGGCGACGCTGCTCCCGGGGGCGAAGGCATTACAGTGTTTCTGCCACCTCTGTACAAAGGATAATTTTACCTGTGAGACAGATGGTCTTTGCTTTGTCTCAGTCACTGAGACCACAGACAAAGTTATACACAATAGTATGTGTATAGCTGAAATTGACCTAATTCCTCGAGACAGGCCATTTGTATGTGCACCATCTTCAAAAACAGGGGCAGTTACTACAACATATTGCTGCAATCAGGACCACTGCAATAAAATAGAACTCCCAACTACAGGACCTTTTTCAGAAAAGCAGTCAGCTGGCCTTGGTCCTGTGGAGCTGGCAGCTGTCATTGCTGGTCCAGTCTGCTTCGTCTGCATTGCACTTATGCTGATGGTCTATATCTGCCATAACCGCACTGTCATTCACCACCGTGTGCCAAATGAAGAGGATCCATCACTAGATCGCCCTTTCATTTCAGAGGGCACCACCTTAAAAGATTTAATTTATGATATGACAACATCAGGGTCTGGATCAGGTTTACCACTGCTTGTTCAAAGAACAATTGCCAGGGACATTGTGTTACAAGAAAGCATTGGCAAAGGTCGGTTTGGAGAAGTTTGGCGAGGCAAATGGCGGGGAGAAGAAGTTGCTGTGAAGATATTCTCTTCTAGAGAAGAGCGTTCATGGTTCCGAGAGGCAGAGATTTATCAGACTGTAATGTTACGCCATGAAAATATCCTGGGATTTATAGCAGCAGACAACAAAGACAATGGGACATGGACGCAGCTGTGGTTGGTGTCAGATTATCATGAGCATGGATCCCTTTTCGATTACTTGAATAGATACACTGTTACTGTGGAAGGAATGATCAAGCTTGCTCTGTCCACAGCAAGTGGTCTTGCCCATCTTCACATGGAGATTGTTGGTACCCAAGGAAAACCAGCTATTGCCCATAGAGATTTGAAATCAAAGAATATCTTGGTGAAGAAAAATGGAACCTGTTGTATTGCAGACTTGGGACTTGCTGTGAGACATGATTCTGCCACAGATACAATTGATATTGCTCCAAACCACAGAGTAGGCACTAAAAGGTACATGGCCCCTGAAGTTCTAGATGATTCCATAAATATGAAACATTTTGAATCCTTCAAACGCGCTGACATCTATGCAATGGGCTTAGTGTTCTGGGAAATTGCTCGACGCTGTTCTATTGGTGGAATCCATGAAGACTATCAGTTGCCTTATTATGATCTTGTACCTTCTGATCCATCGGTTGAAGAAATGAGAAAAGTAGTTTGCGAACAGAAGTTAAGGCCAAATATTCCAAACAGATGGCAGAGCTGTGAGGCCTTGAGAGTGATGGCTAAAATTATGAGAGAATGCTGGTATGCCAATGGAGCAGCAAGGCTGACAGCTTTGCGAATTAAAAAAACATTGTCACAACTCAGCCAACAGGAAGGCATCAAAATGTAA

RNA sequencing (RNA-seq) and data analysis

RNA sequencing analyses as reported previously.^2^ Differential gene expression in the different groups was analyzed by the DESeq2 package in R (1.16.1). Gene set enrichment analysis (GSEA), Gene Ontology and Kyoto Encyclopedia of Genes and Genomes (KEGG) Pathway Enrichment Analysis was employed to identify the enriched pathways in BM ECs from the different groups. A *P* value<0.05 was set as the cutoff for the above statistical analysis.

Quantitative RT-PCR (qPCR)

Total RNA from cultured cells was prepared using a microscale RNA isolation kit (QIAGEN). Reverse transcription was carried out according to the manufacturer's instruction (TaKaRa). RNA expression levels were quantified using SYBR Green qPCR kit (Thermo Fisher Scientific). RNA levels were normalized using 18S as an internal control. The relative mRNA levels of *PTN* (forward primer: 5′-TGAGTGCAAGCAAACCATGA-3′; reverse primer: 5′-TCTTCTGGCATTCGGCATTG-3′), in the cultivated BM ECs with or without TGF-β1 treatment were analyzed. Normalized levels of the *PTN* ratios in the qPCR assays were evaluated through comparisons with the 18S levels(forward primer: 5′- GTAACCCGTTGAACCCCATT-3′; reverse primer: 5′-CCATCCAATCGGTAGTAGCG-3′).

Multiplex Immunohistochemistry (IHC)

For multiplex IHC, OpalPolaris7-ColorManual IHC Kit (NEL861001KT,AKOYA) were used. Tissue slices were deparaffinized with xylene, rehydrated through a descending ethanol series (100%, 95%, 90%, 85%), treated with 3% hydrogen peroxide, and subjected to antigen retrieval using Tris-EDTA buffer (pH 9.0). After blocking with 5% BSA, the slices were incubated with primary antibodies against Sca1 (1:50, 557403, BD Biosciences), Endomucin (1:70, sc-65495, Santa Cruz), and Flag (1:150, ab205606, Abcam) in a humidified chamber at 37 °C for 60 min. They were then incubated with HRP-conjugated secondary antibodies (goat anti-mouse or goat anti-rabbit) at 37 °C for 10 min. Before the next step, redundant antibodies were removed by placing the slides in Tris-EDTA buffer (pH 9.0). Finally, the slices were counterstained with DAPI solution at 37 °C for 10 min in the dark. Images were acquired using the Vectra Polaris Quantitative Pathology Imaging System.

Bioinformatics and statistics

Bioinformatic and statistical analyses were performed using Perseus (version 2.0.11).^3,4^ A *p*-value <0.05 from ANOVA and a fold change of 1.5 defined differential phosphorylation. The Wikipathway analysis was performed with Cytoscape.^5^

Statistical analysis was performed using GraphPad Prism. All data are expressed as the mean ± SEM. The student’s t test was used for two groups with a normal distribution, and the Mann-Whitney U test was used for two groups with a non-normal distribution. A *p* value ≤ 0.05 was considered significant.

The clinical characteristics of patients in the prospective clinical trial

The underlying diseases of patients in this trial were acute myeloid leukemia (N=21), acute lymphoblastic leukemia (N=16), myelodysplastic neoplasm (N=16), aplastic anemia (N=6), chronic myelomonocytic leukemia (N=1), lymphoblastic lymphoma (N=2), mixed-phenotype acute leukemia (N=1) and myelofibrosis (N=1), respectively. The median age of the patients was 46 years (range 14-74 years). Eleven patients received HLA-matched sibling donors, 5 matched unrelated donors, and 48 haploidentical donors. The blood types of 30 (46.9%) patients were matched between the donors and recipients, whereas those of 34 patients were mismatched. Patients received a median of 9.6 (4.4-22.8) × 10^8^/kg mononuclear cells and 3.1 (0.7-8.4) × 10^6^/kg CD34^+^ cells, respectively.

Luspatercept treatment

The dose of luspatercept ranged from 0.7 to 1.4 mg/kg subcutaneously. Appropriate supportive care (including antibiotics, antifungals, and antivirals) was permitted. Luspatercept was initiated on a median of 104 (13-1388) days following allo-HSCT. Adverse events were evaluated following the National Cancer Institute Common Toxicity Criteria version 5.0.

Figure. S1.

**
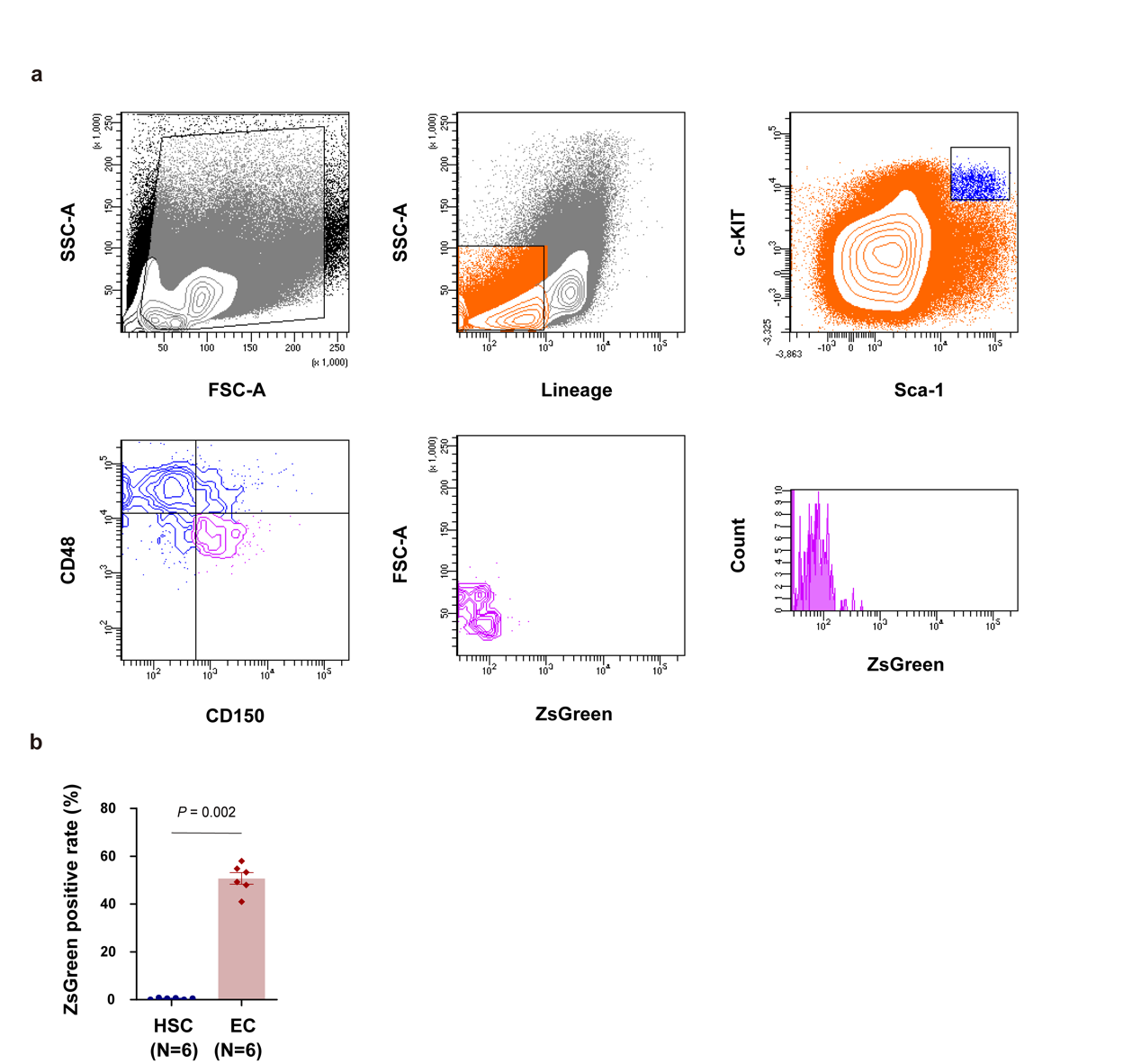
**

**Supplementary Fig. 1 The transduction efficiency of AAV-V_EC_ in vivo.** Adult female C57BL/6J mice (8-10-weeks-old) received a single intraosseous injection of recombinant AAV-VEC. AAV-TGF-βRI mice were administered AAV-VEC encoding TGF-βRI, a Flag tag, and ZsGreen under the control of an endothelial cell (EC)-specific Tie promoter. Gating strategy (**a**) and quantification (**b**) of the percentage of Zsgreen^+^ BM ECs percentage in the total BM ECs (CD45^-^Ter119^-^CD31^+^VE-Cadherin^+^) and Zsgreen^+^ HSCs in the total HSCs (Lineage^-^cKIT^+^SCA1^+^CD150^+^CD48^-^) from mice with indicated treatment in steady-state conditions by flow cytometry (N= 6 per group, *n* = 1 per sample). N represents biological replicates; *n* represents technical replicates. Statistical analyses were performed using Mann-Whitney U test. The data are presented as the means ± SEMs. SEM: standard error of the mean.

Figure. S2.

**
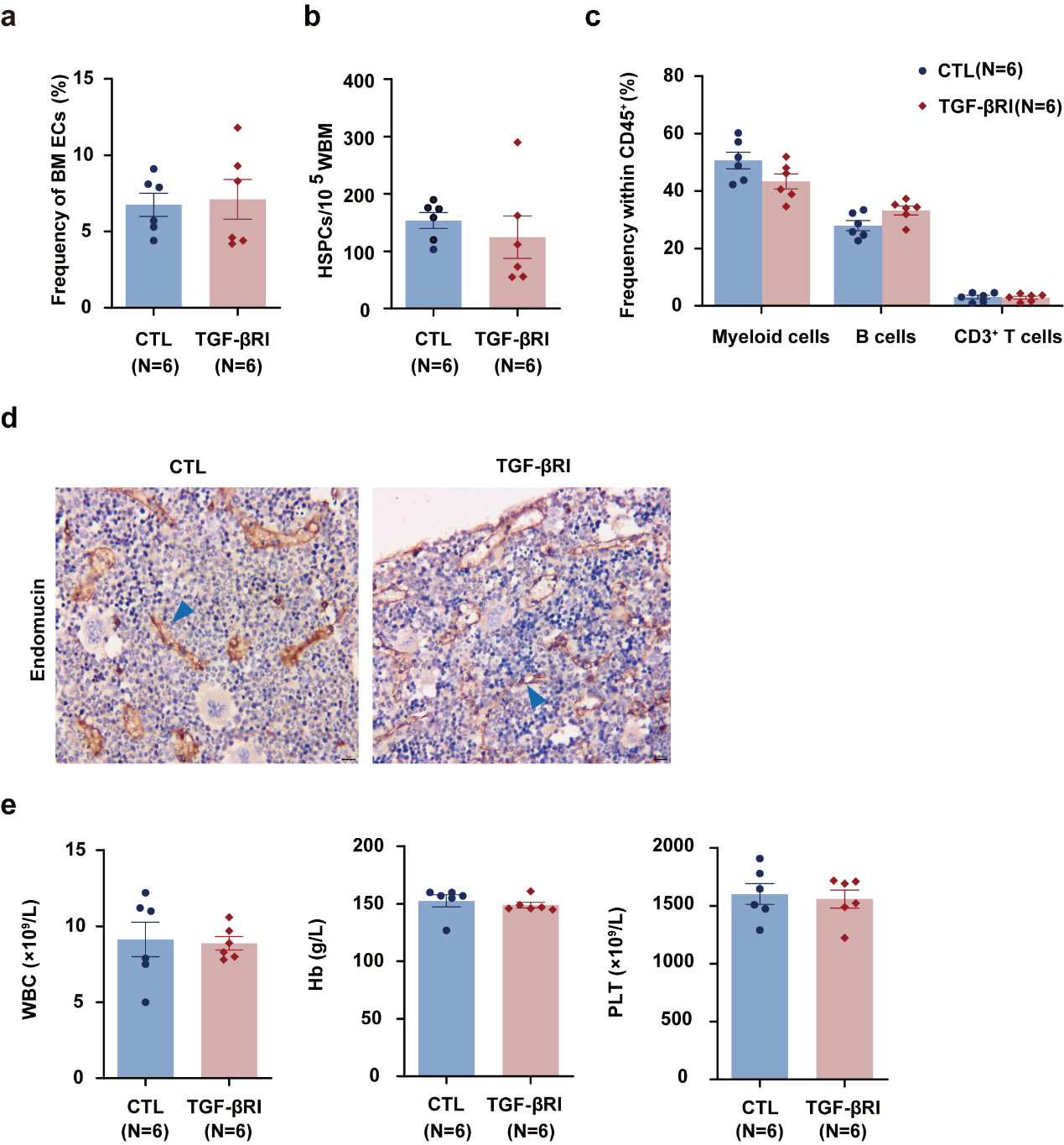
**

**Supplementary Fig.** **2 Establishment of a BM EC-specific TGF-βRI overexpression murine model.** Adult female C57BL/6J mice (8-10-weeks-old) were administered a single dose of recombinant AAV-V_EC_ via intraosseous injection. AAV-TGF-βRI mice (TGF-βRI) received AAV-V_EC_ encoding the TGF-βRI, Flag tag and ZsGreen genes under the control of an endothelial cell (EC)-specific Tie promoter. TGF-βRI (T204D) indicates that the threonine at position 204 is replaced by aspartic acid, mimicking the persistent activation of TGF-βRI receptor. AAV-CTL mice (CTL), which were age- and sex-matched, received AAV-V_EC_ encoding only the Flag tag and ZsGreen genes under the control of the same Tie promoter. **a-c** The percentages of CD31^+^VE-Cadherin^+^ ECs within the BM CD45^-^Ter119^-^ cells (**a**), HSPCs (Lineage^-^c-KIT^+^SCA1^+^) in whole BM cells (WBM) (**b**), and lineage committed hematopoietic cells within the BM CD45^+^ cells (**c**) from CTL mice and TGF-βRI mice in the steady-state condition (N= 6 per group, *n* = 1 per sample). **d** Anti-Endomucin antibody stained femur sections showed representative BM ECs in the steady-state condition. Scale bar=10 μm. Normal (blue arrowhead) vessels are noted. **e** Peripheral blood (PB) cell counts, including white blood cells (WBCs), hemoglobin (Hb), and platelets (PLT), were measured from CTL mice and TGF-βRI mice in the steady-state condition (N= 6 per group, *n* = 3 per sample). N represents biological replicates; *n* represents technical replicates. Statistical analyses were performed using the Mann-Whitney U test. The data are presented as the means ± SEMs. SEM: standard error of the mean.

Figure. S3.


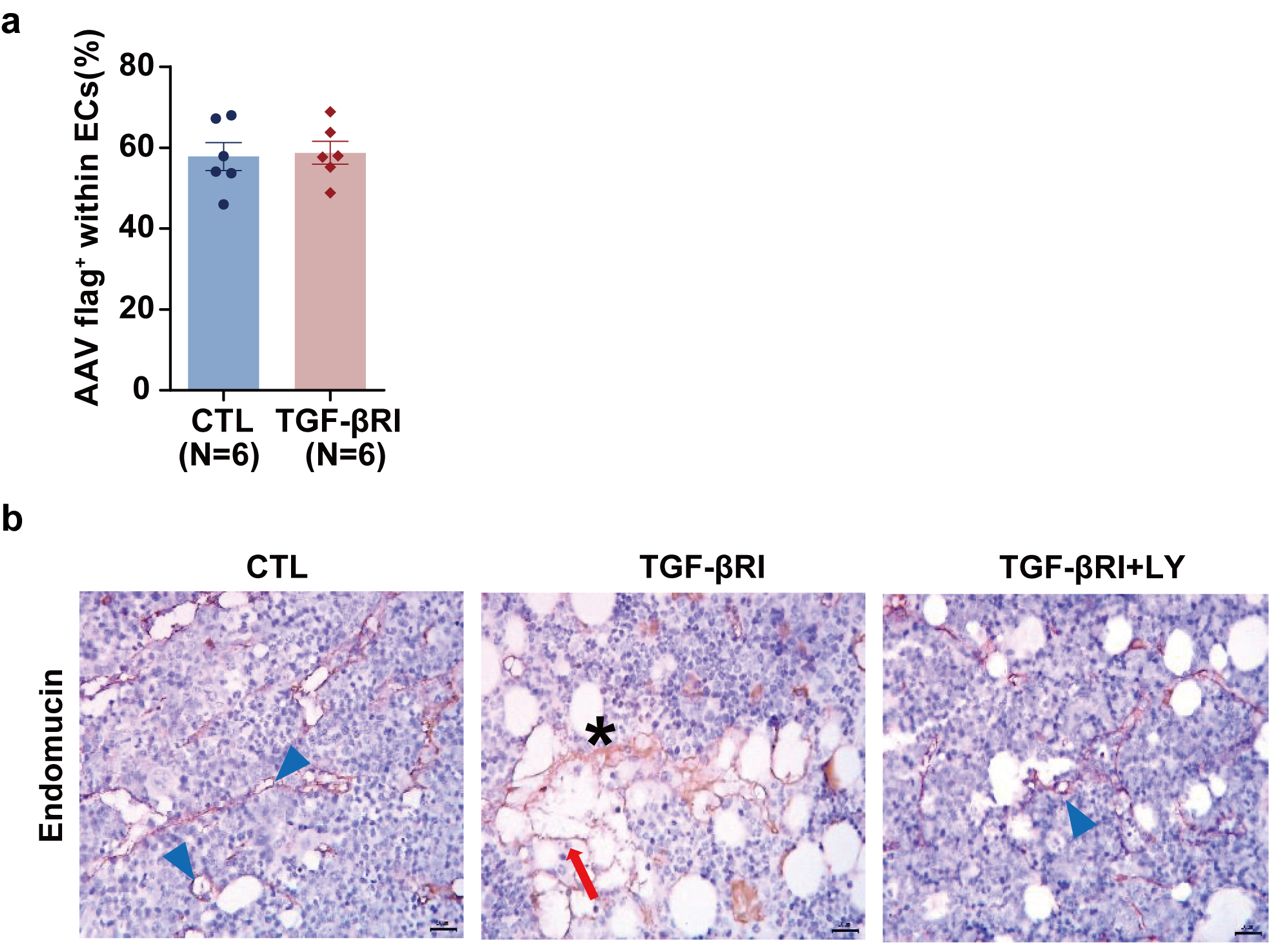


**Supplementary Fig. 3 BM EC-specific TGF-βRI overexpression impaired BM vessel structures.** Cohorts of mice were treated with 5-fluorouracil (5-FU) at a dose of 250 mg/kg via tail vein injection on day 0. Subsequently, they were administered LY2157299 (LY) or dimethyl sulfoxide (DMSO) control daily via intragastric gavage. **a** Quantification of Flag^+^ BM ECs percentage in the total BM ECs (CD45^-^Ter119^-^CD31^+^VE-Cadherin^+^) from mice with indicated treatment were analyzed by flow cytometry (N= 6 per group, *n* = 1 per sample). **b** Representative images demonstrate pathological alterations in BM ECs within murine femoral sections immunostained with anti-Endomucin antibody on day 14. Scale bars represent 10 µm. Normal (blue arrowhead), dilated (red arrow) and dilated and discontinuous (black asterisk) vessels were noted. N represents biological replicates; *n* represents technical replicates. Statistical analyses were performed using the Mann-Whitney U test. The data are presented as the means ± SEMs. SEM: standard error of the mean.

Figure. S4.


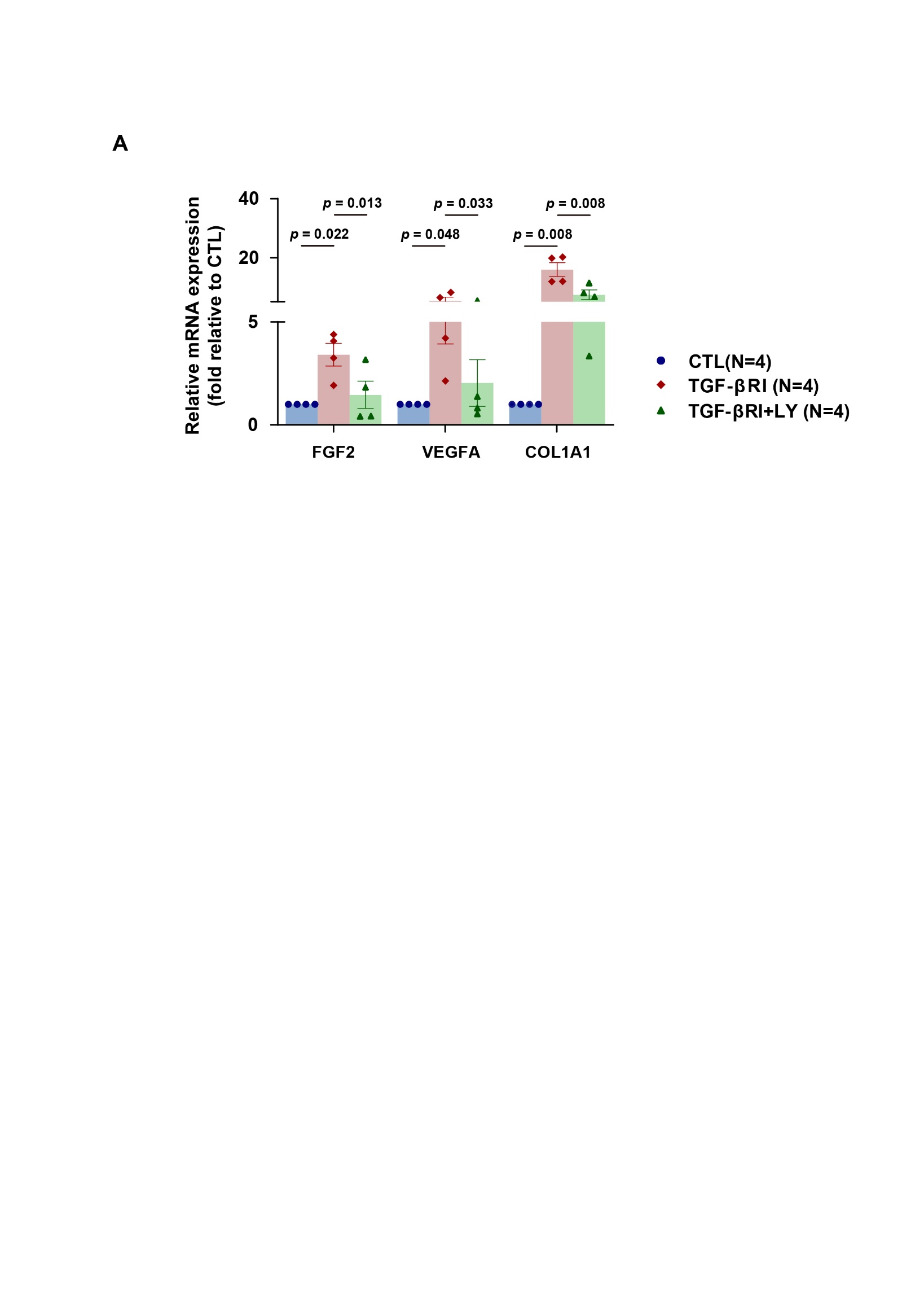


**Supplementary Fig. 4** The mRNA levels of FGF2, VEGFA, COL1A1 in BM ECs treated with TGF-β1 alone or in combination with LY2157299 (LY) were assessed via qPCR (N= 4 per group, *n* = 1 per sample). N represents biological replicates; *n* represents technical replicates. Statistical analyses were performed using the paired t test. The data are presented as the means ± SEMs. SEM: standard error of the mean.

Figure. S5.

**
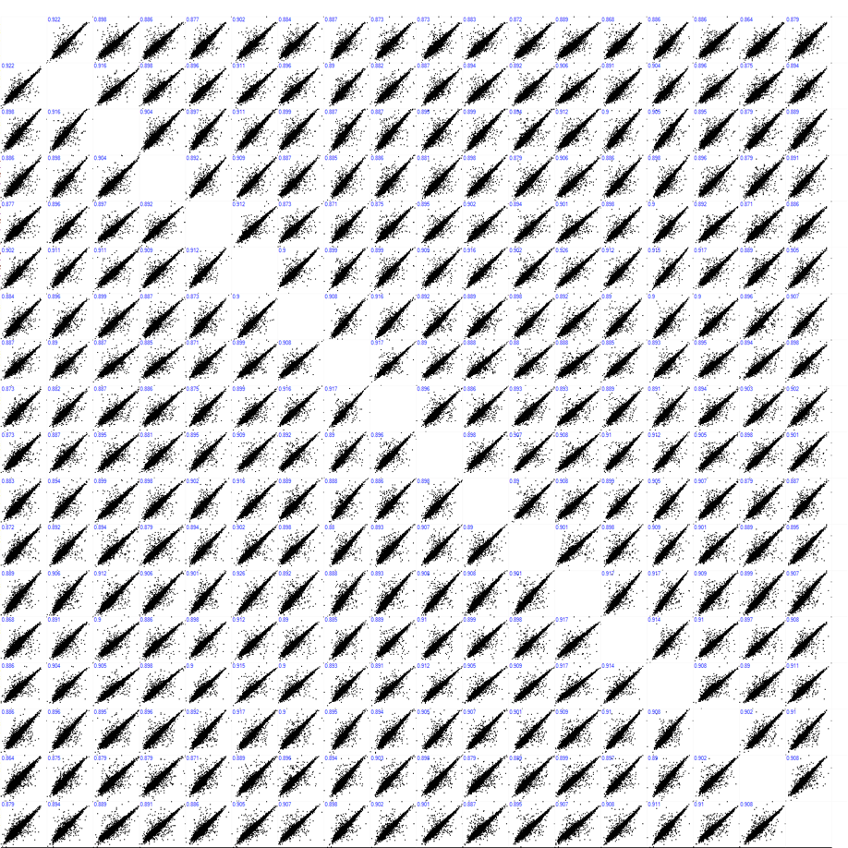
**

**Supplementary Fig. 5** The multi-scatterplot shows the result of the Pearson correlation analysis of the log10-transformed intensities of phosphopeptides in three biological replicates.

Figure. S6.

**
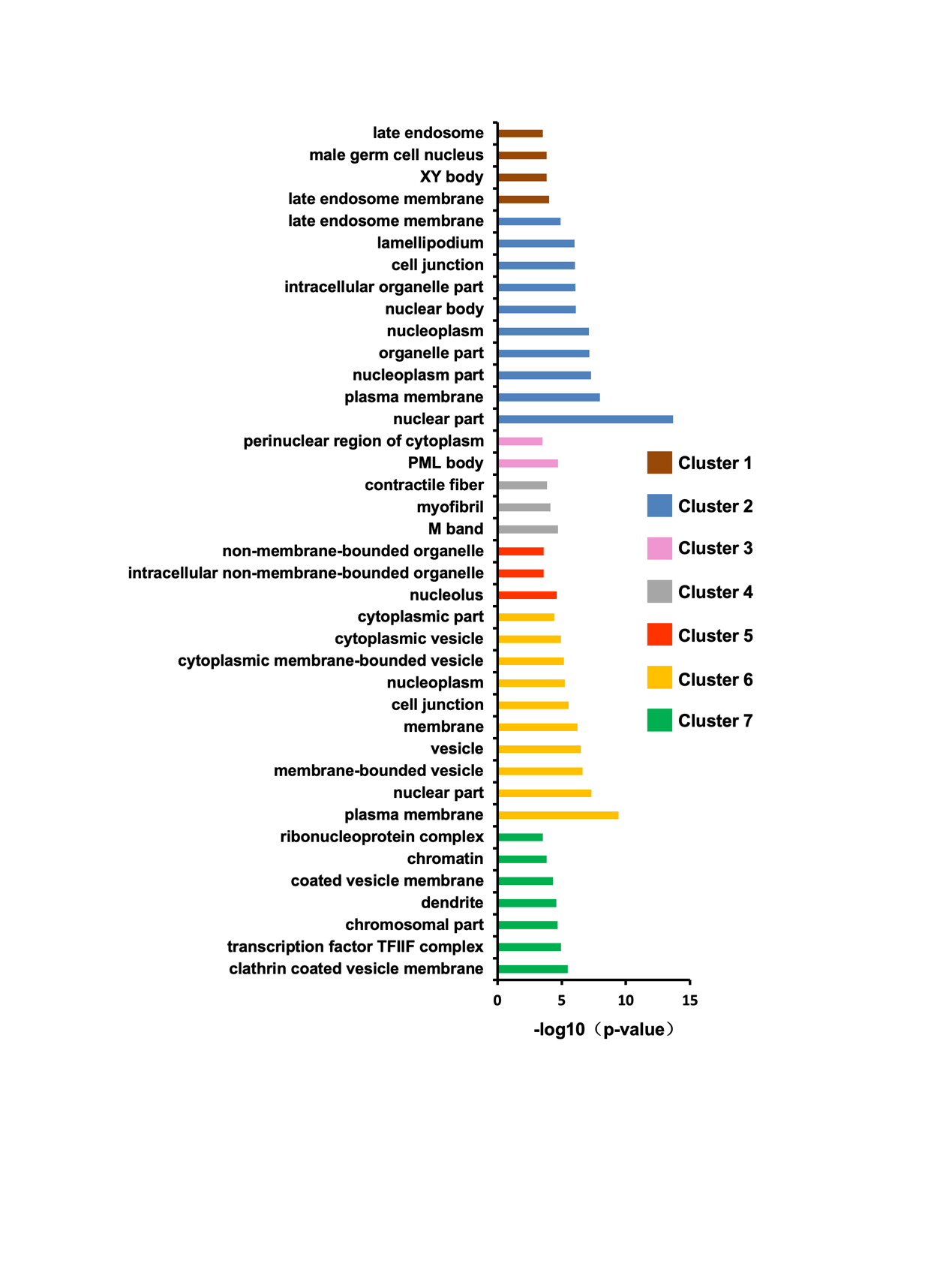
**

**Supplementary Fig. 6** The bar graph shows the GO terms (cellular component) enriched in the phosphoproteins identified from 7 clusters. The top ten GO terms ranked by decreasing -log10 (*p*-value) are displayed.

Figure. S7.

**
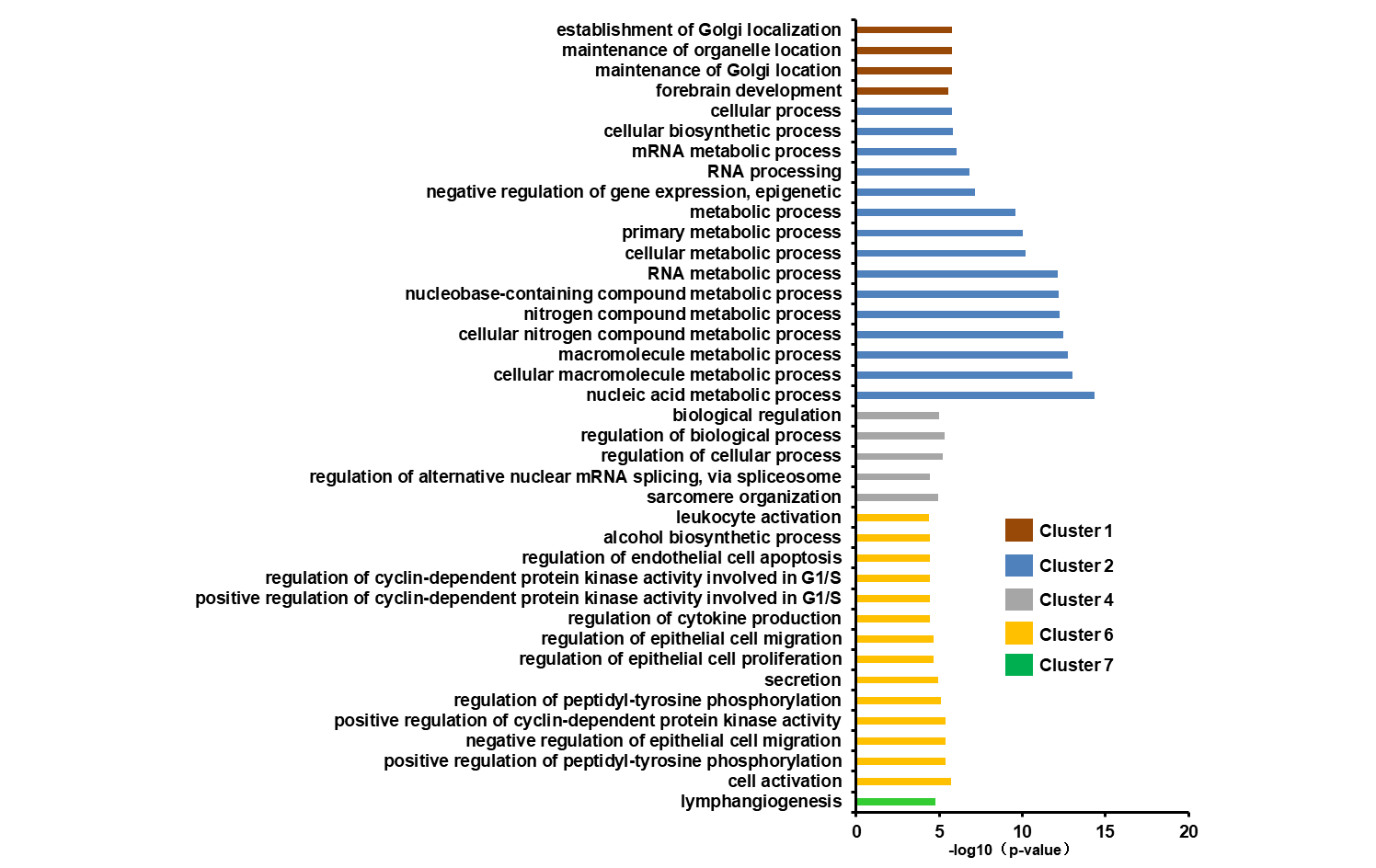
**

**Supplementary Fig. 7** The bar graph shows the GO terms (biological progress) enriched in the phosphoproteins identified from 7 clusters. The top 15 GO terms ranked by decreasing -log10 (*p*-value) are displayed.

Figure. S8.

**
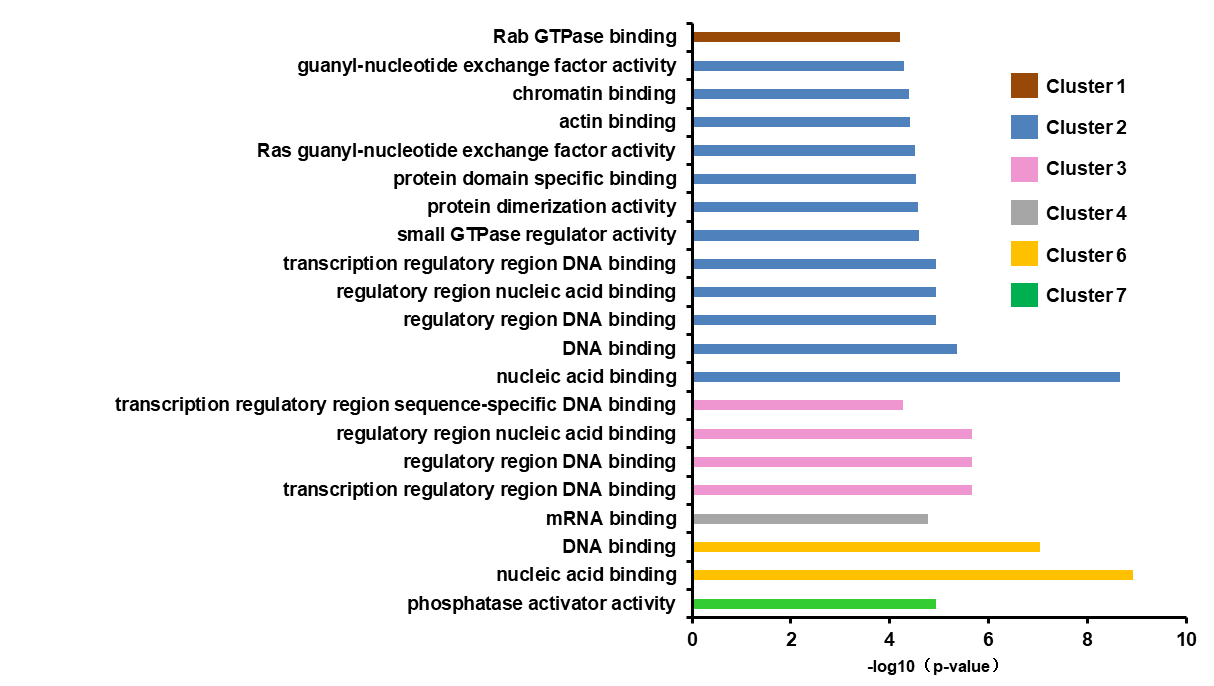
**

**Supplementary Fig. 8** The bar graph shows the GO terms (molecular function) enriched in the phosphoproteins identified from 7 clusters. The all GO terms ranked by decreasing -log10 (*p*-value) are displayed.

Figure. S9.

**
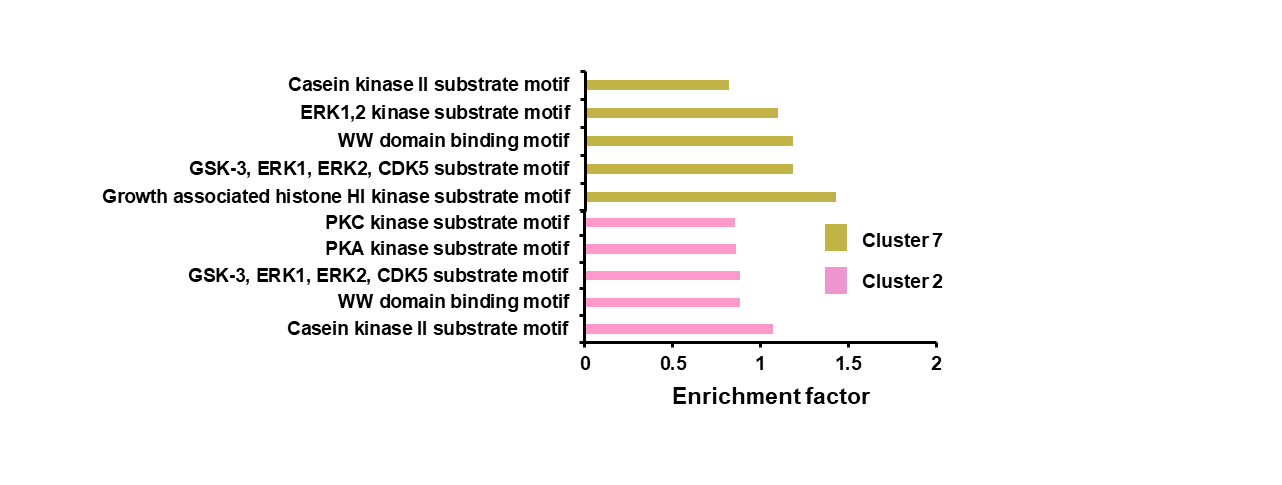
**

**Supplementary Fig. 9** Fisher’s exact test on linear motif with clustered phosphosites upon by Perseus.

Figure. S10.

**
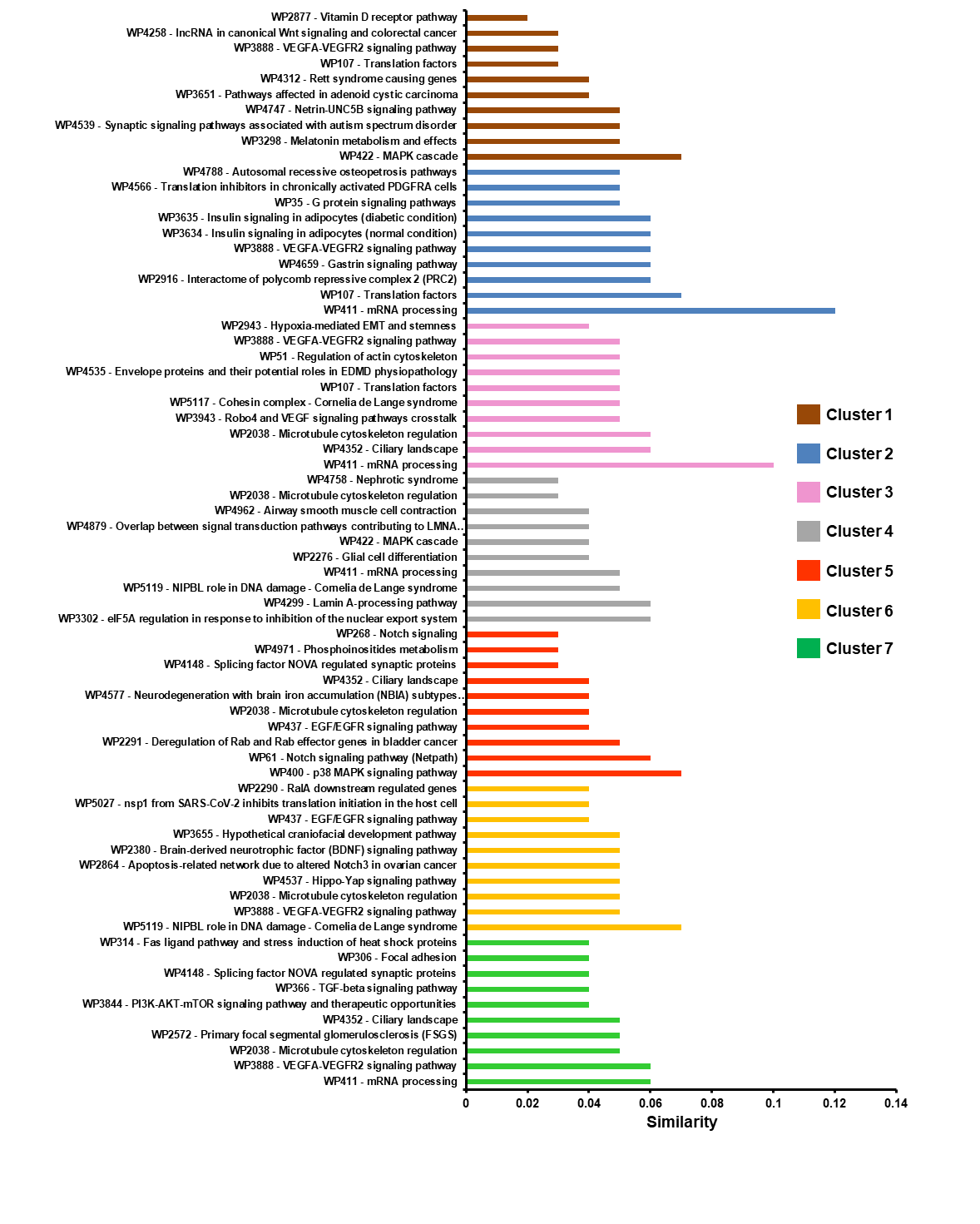
**

**Supplementary Fig. 10** The bar graph shows the Wikipathways enriched in the phosphoproteins identified from 7 clusters. The top ten similarity ranked by decreasing -log10 (*p*-value) are displayed.

Figure. S11.


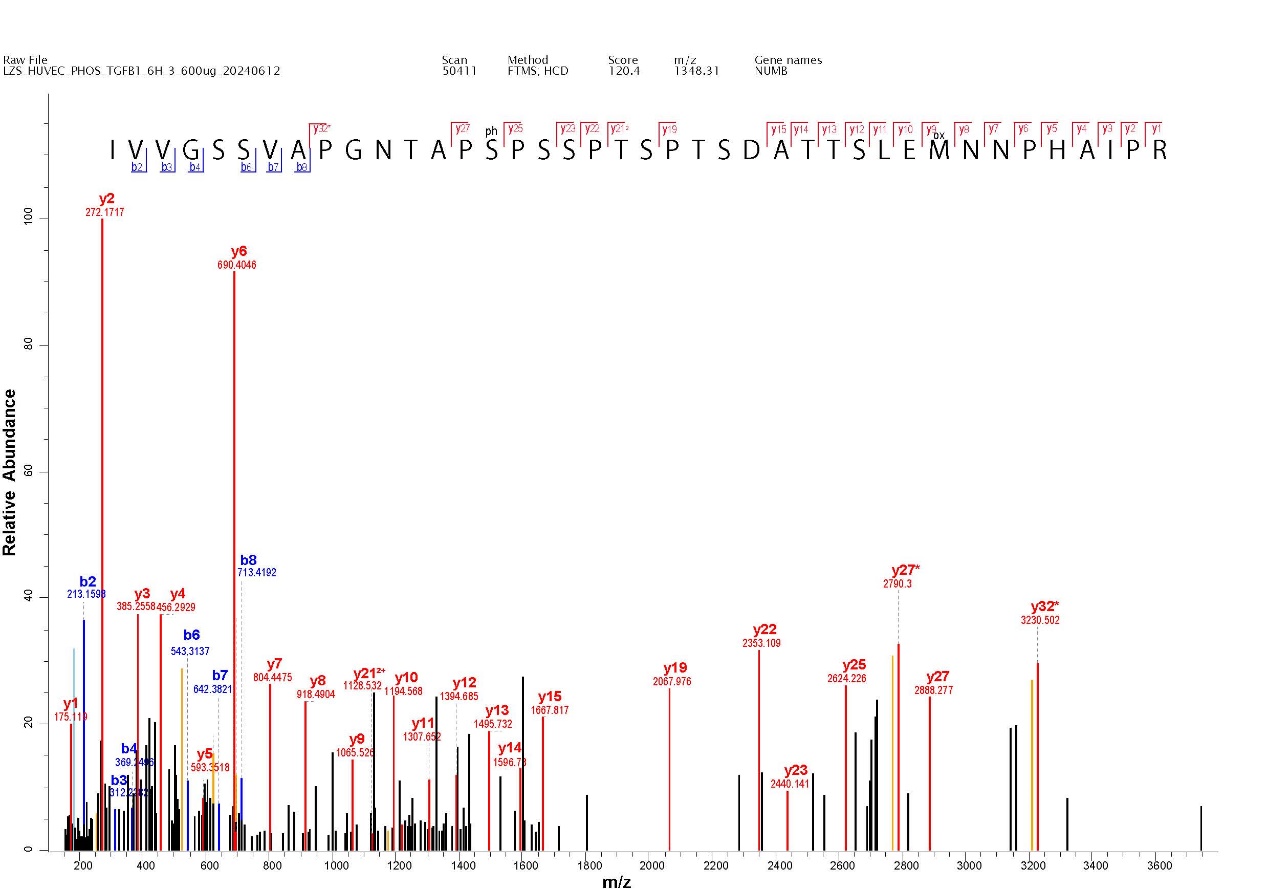


**Supplementary Fig. 11** The mass spectrum of peptide containing phosphorylated S227 from NUMB. The amino acid sequence is shown with the modified site marked by ph. The matched b- and y-ions are labeled, confirming the peptide sequence. The neutral loss of phosphoric acid (H₃PO₄, 98 Da) is indicated by an asterisk.

Figure. S12.


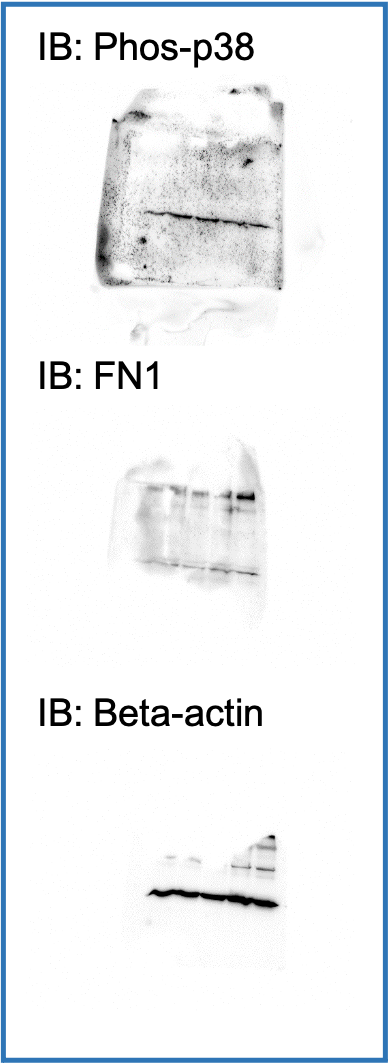


**Supplementary Fig. 12** Raw gel data from Figure 5g.

Figure. S13.

**
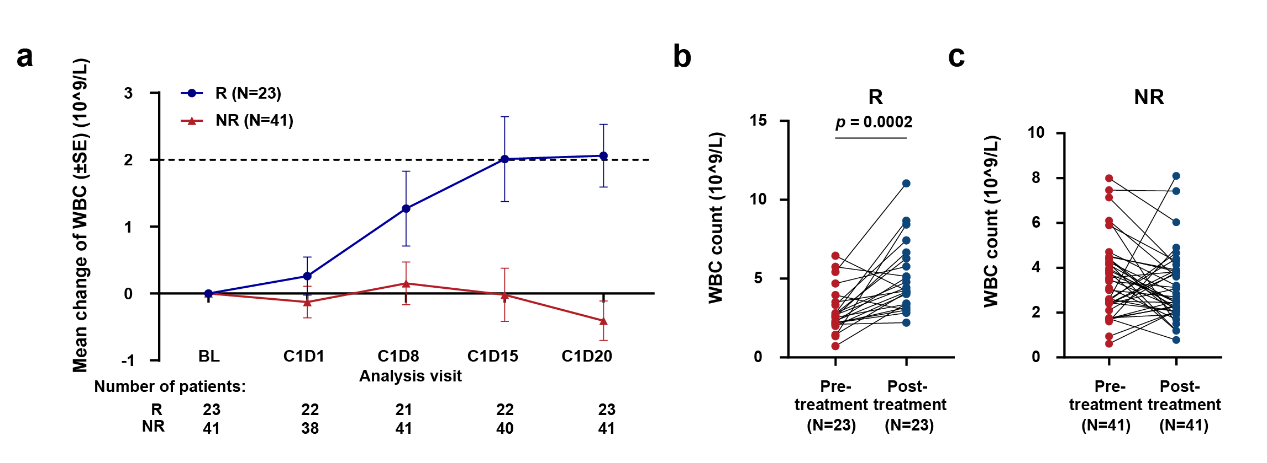
**

**Supplementary Fig. 13 a** The mean change in the levels of WBC counts over time in patients had a response (R) and in those who did not have a response (NR). **b-c** The observed levels of WBC counts pre-treatment and post-treatment in R (**b**) and NR (**c**) groups, respectively. WBC, white blood cell.

Table S1. Characteristics of patients with PGF and GGF post allo-HSCT

| **Characteristics** | **PGF (N=15)** | **GGF (N=15)** | ***p*-value** |
| --- | --- | --- | --- |
| **BM evaluated time**  (post-HSCT days, median, range) | 60 (30-180) | 90 (30-180) | 0.382 |
| **Blood cell count** |  |  |  |
| Median WBC (×10^9^/L) (range) | 2.40 (0.75-5.80) | 3.70 (1.48-10.60) | 0.049 |
| Median ANC (×10^9^/L) (range) | 1.40 (0.00-5.10) | 1.70 (0.60-9.10) | 0.254 |
| Median Hb (×g/L) (range) | 72 (64-91) | 105 (80-134) | <0.0001 |
| Median PLT (×10^9^/L) (range) | 19 (10-47) | 104 (32-193) | <0.0001 |
| **Age at HSCT**  (years, median, range) | 29 (11-54) | 35 (15-54) | 0.926 |
| **Gender** (male/female) | 7/8 | 8/7 | 0.715 |
| **Underlying disease** |  |  | >0.9999 |
| AML | 5 | 5 |  |
| ALL | 5 | 5 |  |
| MDS | 5 | 5 |  |
| **Status at HSCT** |  |  | 1.000 |
| Standard-risk | 15 | 15 |  |
| High-risk | 0 | 0 |  |
| **Source of stem cell** |  |  | 0.269 |
| PB | 5 | 8 |  |
| BM and PB | 10 | 7 |  |
| **Transplanted total nucleated cell dose** (×10^8^/kg, median, range) | 8.26 (6.44-11.23) | 7.89 (5.10-10.51) | 0.091 |
| **Transplanted CD34^+^ cell dose** (×10^6^/kg, median, range) | 2.08 (1.30-3.42) | 2.67 (1.06-8.03) | 0.205 |
| **Donor match** |  |  | 0.549 |
| HLA-identical sibling donor | 3 | 2 |  |
| HLA-identical unrelated donor | 0 | 1 |  |
| HLA-partially matched related donor | 12 | 12 |  |
| **Sex mismatch** |  |  | 0.145 |
| Female to male | 4 | 1 |  |
| Male to female | 6 | 4 |  |
| No | 5 | 10 |  |
| **ABO mismatch** |  |  | 0.436 |
| No | 13 | 10 |  |
| Major | 1 | 2 |  |
| Minor | 0 | 2 |  |
| Bidirectional | 1 | 1 |  |
| **Pre-HSCT cycles of chemotherapy** | 3 (0-5) | 3 (0-7) | 0.924 |
| **Conditioning** |  |  | 0.223 |
| BU/CY | 3 | 1 |  |
| BU/CY+ATG | 12 | 12 |  |
| BU/CY/Flu+ATG | 0 | 2 |  |
| **History of CMV reactivation** | 4 | 3 | 0.666 |
| Onset of CMV reactivation  (days, median, range) | 16 (5-42) | 11 (4-21) | 0.600 |
| **History of aGVHD** | 8 | 5 | 0.269 |
| Onset of aGVHD  (days, median, range) | 21 (7-53) | 22 (6-51) | 0.971 |
|  |  |  |  |
| The continuous variables were compared using the Mann-Whitney U-test, and the differences in frequency between the 2 groups were compared using the chi-square test.  The criterion for statistical significance was *p*<0.05.  **Abbreviations:** allo-HSCT indicates allogeneic hematopoietic stem cell transplantation; aGVHD, acute graft-versus-host disease; Flu, fludarabine; PGF, poor graft function; GGF, good graft function; BM, bone marrow; PB, peripheral blood; WBC, white blood cell; ANC, absolute neutrophil cell; Hb, hemoglobin; PLT, platelet; AML, acute myelogenous leukemia; ALL, acute lymphocytic leukemia; MDS, myelodysplastic syndrome; HLA, human leukocyte antigen; BU/CY, busulfan, cyclophosphamide and cytarabine; CMV, cytomegalovirus. | | | |

References

1. Korhonen, J. et al. Endothelial-specific gene expression directed by the tie gene promoter in vivo. *Blood* **86**, 1828-1835 (1995).

2. Chen, J. et al. Spatial transcriptomic analysis of cryosectioned tissue samples with Geo-seq. *Nat Protoc* **12**, 566-580 (2017).

3. Tyanova, S. et al. The Perseus computational platform for comprehensive analysis of (prote)omics data. *Nat Methods* **13**, 731-740 (2016).

4. Tyanova, S., Cox, J. Perseus: A Bioinformatics Platform for Integrative Analysis of Proteomics Data in Cancer Research. *Methods Mol Biol* **1711**, 133-148 (2018).

5. Agrawal, A. et al. WikiPathways 2024: next generation pathway database. *Nucleic Acids Res* **52,** D679-D689 (2024).
